# Functional Analysis of the α-1,3-Glucan Synthase Genes *agsA* and *agsB* in *Aspergillus nidulans*: AgsB Is the Major α-1,3-Glucan Synthase in This Fungus

**DOI:** 10.1371/journal.pone.0054893

**Published:** 2013-01-24

**Authors:** Akira Yoshimi, Motoaki Sano, Azusa Inaba, Yuko Kokubun, Tomonori Fujioka, Osamu Mizutani, Daisuke Hagiwara, Takashi Fujikawa, Marie Nishimura, Shigekazu Yano, Shin Kasahara, Kiminori Shimizu, Masashi Yamaguchi, Kazuyoshi Kawakami, Keietsu Abe

**Affiliations:** 1 New Industry Creation Hatchery Center, Tohoku University, Sendai, Japan; 2 Genome Biotechnology Laboratory, Kanazawa Institute of Technology, Ishikawa, Japan; 3 Department of Microbial Biotechnology, Tohoku University, Sendai, Japan; 4 Kumiai Chemical Industry Co., Ltd., Tokyo, Japan; 5 National Research Institute of Brewing, Higashi-Hiroshima, Japan; 6 Medical Mycology Research Center, Chiba University, Chiba, Japan; 7 National Institute of Agrobiological Sciences, Tsukuba, Japan; 8 Department of Biotechnology, Ritsumeikan University, Kusatsu, Japan; 9 Department of Environmental Sciences, Miyagi University, Sendai, Japan; 10 National Department of Medical Microbiology, Tohoku University, Sendai, Japan; University of Wisconsin - Madison, United States of America

## Abstract

Although α-1,3-glucan is one of the major cell wall polysaccharides in filamentous fungi, the physiological roles of α-1,3-glucan remain unclear. The model fungus *Aspergillus nidulans* possesses two α-1,3-glucan synthase (AGS) genes, *agsA* and *agsB*. For functional analysis of these genes, we constructed several mutant strains in *A. nidulans*: *agsA* disruption, *agsB* disruption, and double-disruption strains. We also constructed several CagsB strains in which *agsB* expression was controlled by the inducible *alcA* promoter, with or without the *agsA-*disrupting mutation. The *agsA* disruption strains did not show markedly different phenotypes from those of the wild-type strain. The *agsB* disruption strains formed dispersed hyphal cells under liquid culture conditions, regardless of the *agsA* genetic background. Dispersed hyphal cells were also observed in liquid culture of the CagsB strains when *agsB* expression was repressed, whereas these strains grew normally in plate culture even under the *agsB*-repressed conditions. Fractionation of the cell wall based on the alkali solubility of its components, quantification of sugars, and ^13^C-NMR spectroscopic analysis revealed that α-1,3-glucan was the main component of the alkali-soluble fraction in the wild-type and *agsA* disruption strains, but almost no α-1,3-glucan was found in the alkali-soluble fraction derived from either the *agsB* disruption strain or the CagsB strain under the *agsB*-repressed conditions, regardless of the *agsA* genetic background. Taken together, our data demonstrate that the two AGS genes are dispensable in *A. nidulans*, but that AgsB is required for normal growth characteristics under liquid culture conditions and is the major AGS in this species.

## Introduction

The fungal cell wall is a complex structure that is essential for the maintenance of cellular shape and integrity, prevention of cell lysis, and protection against adverse environmental conditions. Fungal cells constantly remodel their rigid structure during growth and development. Proper cell wall architecture requires several cell wall components that are mainly composed of polysaccharides: α-glucans (α-1,3-glucan and α-1,4-glucan, which are polysaccharides composed of D-glucose), β-glucans (β-1,3-glucan and β-1,6-glucan), mannan, and chitin [1]–[4]. Because of the biological importance and structural specificity of the fungal cell wall, inhibitors that affect the fungal cell wall have been considered ideal drugs against both mammalian- and plant-pathogenic fungi for many years. Indeed, the group of β-1,3-glucan synthase inhibitors called “echinocandins”, which include anidulafungin, caspofungin, and micafungin, have been used commercially to control a variety of fungal pathogens [5].

Cell wall biogenesis and its regulation by cell signaling have been well characterized in the yeast *Saccharomyces cerevisiae* [6], [7]. The activation of the cell wall integrity signaling (CWIS) pathway leads to activation of the downstream mitogen-activated protein kinase (MAPK) cascade via Pkc1p and the small G protein Rho1p, which acts upon Pkc1p [8]. The MAPK Mpk1 protein activates the transcription factor Rlm1p, which regulates the transcription of at least 25 genes involved in cell wall biogenesis, including β-1,3-glucan synthase genes and chitin synthase genes [9]. In the model filamentous fungus *Aspergillus nidulans*, Fujioka et al. [10] constructed disruptants of *mpkA* and *rlmA* (orthologues of *S. cerevisiae MPK1* and *RLM1*, respectively), as well as disruptants of *Answi4* and *Answi6* (orthologues of *S. cerevisiae SWI4* and *SWI6*, which together encode the Mpk1p-activating transcription factor Swi4p–Swi6p complex). The transcription of most cell wall–related genes, except for two α-1,3-glucan synthase (AGS) genes (*agsA* and *agsB*), is transiently up-regulated in these mutants by treatment with the β-1,3-glucan synthase inhibitor micafungin; thus, transcription of the β-1,3-glucan synthase gene *fksA* and of several chitin synthase genes is independent of RlmA and AnSwi4/AnSwi6, suggesting that their transcription is regulated by a non-MpkA pathway. The transcription of *agsA* is weakly up-regulated in *mpkA*Δ and *rlmA*Δ strains, and the transcription of *agsB* depends mainly on MpkA–RlmA signaling [10]. Based on these results, Fujioka et al. [10] concluded that the transcriptional regulation of the α-1,3-glucan synthase genes *agsA* and *agsB* was specifically regulated by the MpkA pathway in *A. nidulans*, and that regulation of cell wall–related genes via CWIS in *A. nidulans* differed markedly from that in *S. cerevisiae* [10].

Among the *Aspergillus* species, the functions of α-1,3-glucan synthase genes have been studied in *A. fumigatus* and *A. niger* [11]–[14]. *Aspergillus fumigatus* contains three AGS genes, *ags*1 to *ags*3. *Aspergillus fumigatus ags1*, which is an orthologue of *A. nidulans agsB*, is involved in the formation of 50% of the cell wall α-1,3-glucan, whereas disruption of *ags2*, which is an orthologue of *agsA*, had no detectable effect on glucan levels [11]. Disruption of the third gene, *ags3*, which has no orthologue in *A. nidulans*, results in overexpression of *ags1*, which may serve to compensate for the lost enzyme activity and maintain normal cell wall composition [12]. In addition, disruption of *ags3* in *A. fumigatus* causes hypervirulence, whereas the disruption of *ags1* and *ags2* did not affect virulence [11], [12]. *A. niger* has five α-1,3-glucan synthases encoded by *agsA* to *agsE*. The expression of *agsA* (an orthologue of *A. fumigatus ags3*) and *agsE* (an orthologue of *A. fumigatus ags1* and *A. nidulans agsB*) was induced in the presence of cell wall stress–inducing compounds such as calcofluor white (CFW), sodium dodecyl sulfate, and caspofungin [13]. More recently, a triple-mutant strain of *A. fumigatus* lacking the three α-1,3-glucan synthase genes (*ags1*, *ags2*, and *ags3*) was generated, and growth of the triple mutant in plate culture was similar to that of the parental strain [14]. The triple mutant showed slightly decreased conidiogenesis, as did the single *ags1* and *ags2* mutants [11], [14], and the lack of cell wall α-1,3-glucan led to an increase in β-1,3-glucan and chitin levels in mycelia of the triple mutant [14]. Despite these studies of the functions of α-1,3-glucan synthase genes, the biological roles of α-1,3-glucan are only partly understood.

The importance of cell wall α-1,3-glucan in relation to fungal virulence has been studied in several pathogenic fungi. In the opportunistic pathogen *Cryptococcus neoformans*, loss of cell wall α-1,3-glucan leads to loss of the surface capsule [15]. The capsule appears to promote pathogenesis in the wild-type strain, since mutants that lack cell wall α-1,3-glucan are unable to grow in a mouse model of infection [16]. A correlation between reduction in α-1,3-glucan levels and virulence has also been observed in several dimorphic fungal pathogens, including *Histoplasma capsulatum*, *Paracoccidioides brasiliensis*, and *Blastomyces dermatitidis* [17], [18], [19]. Rappleye et al. [20] reported that α-1,3-glucan conceals β-glucans on the cell wall of *H. capsulatum* from dectin-1-mediated detection by immune cells, blocking host recognition of fungal invasion. Rappleye et al. [20] assumed that pathogenic fungi have evolved such unique stealth mechanisms because they conceal the immunostimulatory molecular patterns of the pathogen from recognition via leukocyte receptors, which disrupts host immune responses. In the rice blast fungus *Magnaporthe grisea*, α-1,3-glucan masks β-1,3-glucan and chitin in the cell wall of the infectious hyphae; in this way, α-1,3-glucan protects the fungal cell wall from digestive enzymes produced by plant cells during infection and inhibits recognition by host cells [21]. Moreover, expression of the *MgAGS1* AGS gene of *M. grisea* depends fully on Mps1p MAPK, which is an orthologue of Mpk1p in *S. cerevisiae*, whereas the expression of genes that encode other cell wall–related enzymes, such as β-1,3-glucan synthase and chitin synthase, is Mps1p-independent in *M. grisea* [21], as is observed in *A. nidulans* [10]. Thus, the regulation of α-1,3-glucan biosynthesis by the CWIS pathway appears to be conserved in filamentous fungi.

In the present study, we carried out functional analysis of the two α-1,3-glucan synthase genes in *A. nidulans*: *agsA* and *agsB*. We constructed disruption strains of *agsA* and *agsB* (the DagsA and DagsB strains, respectively), an *agsA agsB* double-disruption mutant (the DagsA-DagsB strain), and a conditional-*agsB* strain (CagsB) in which *agsB* expression is conditionally regulated by the *alcA* promoter. We also created a strain in which CagsB was combined with a disruption of *agsA* (the CagsB-DagsA strain). Using these strains, we performed transcriptional analysis of cell wall–related genes and biochemical analyses of cell wall components. These analyses revealed that almost all of the cell wall α-1,3-glucan was lost in the *agsB* disruption mutants, regardless of the presence or absence of *agsA*. We also observed that most of the cell wall α-1,3-glucan was lost in the CagsB strain under *agsB*-repressing conditions, regardless of the *agsA* genetic background. Based on these results, we discuss the relationship of the biological roles of α-1,3-glucan and the two AGS genes in *A. nidulans.*

## Materials and Methods

### Strains and Growth Media

Throughout this study, *A. nidulans* ABPU1 (*biA1, pyrG89, wA3, argB2, pyroA4*) with *ligD*Δ (*ligD*Δ::*ptrA*) was used for all genetic manipulations and for the control strain (CNT). For the *A. nidulans agsA* and *agsB* disruption studies, ABPU1 cells were transformed with the gene replacement constructs described in the following section. The *alcA* promoter–driven *agsB* allele was introduced by transformation of ABPU1 with a replacement cassette containing the *pyrG* marker. Czapek-Dox (CD) medium was used for standard culture [10]. CD medium containing 100 mM threonine and 0.1% fructose instead of 1% glucose as a carbon source (designated CDTF medium henceforth) was used for up-regulation of *alcA* promoter–driven *agsB* [22]. To investigate gene expression, conidia (5×10^5^ cells/mL) were inoculated into liquid medium (CD or CDTF) and cultured at 37°C for 24 h, and then mycelia were harvested. DNA extraction, RNA extraction, reverse transcriptase reactions, and transformation analysis were all performed as described previously [23].

### Construction of Gene Replacement Cassettes

The gene replacement cassettes for the *agsA* and *agsB* disruption mutants (DagsA and DagsB) and the *alcA* promoter–driven *agsB* (CagsB) strain were constructed by PCR fusion following a strategy similar to that used by Izumitsu et al. [24]. The sequences of all primers used in this study are listed in Table S1. To disrupt a gene in *A. nidulans*, we replaced it with an *A. oryzae pyrG* or *argB* gene as the selectable marker. Fungal transformations were performed as described previously [10].

To construct the disruption cassette for the *agsA* gene, the first round of PCR amplified gene fragments containing the 5′ non-coding region (amplicon 1) and the coding region (amplicon 2) of *agsA* from the *A. nidulans* ABPU1 genomic DNA template, and amplified the *pyrG* gene (amplicon 3) from the *A. oryzae* genomic DNA template (Figure S1). Amplicon 1 was amplified with the primers agsA-LU and agsA-LL, amplicon 2 with agsA-RU and agsA-RL, and amplicon 3 with agsA-PU and agsA-PL. The primers agsA-LL, agsA-RU, agsA-PU, and agsA-PL were chimeric oligonucleotides; each contained a reverse-complement sequence for PCR fusion. The three resulting PCR products were gel-purified and used as substrates for a second round of PCR using the primers agsA-LU and agsA-RL to fuse the three separate fragments from the first round into a disruption cassette. All PCR reactions were performed using the GeneAmp PCR System 9700 (Applied Biosystems, Foster City, CA, USA) using PrimeSTAR HS DNA polymerase (Takara, Tokyo, Japan). The resulting major PCR product was gel-purified and used to transform the ABPU1 strain.

To construct the disruption cassette for *agsB*, we used the same PCR fusion strategy. In the first round of PCR, the primers agsB-LU and agsB-LL (for amplicon 1), agsB-RU and agsB-RL (for amplicon 2), and argB-F and argB-R (for amplicon 3) were used (Table S1). The primers agsB-LU and agsB-RL were used for the second round of PCR. The resulting major PCR product was gel-purified and used to transform the ABPU1 strain (Figure S2).

To construct the gene replacement cassette for the *alcA* promoter–driven *agsB* (CagsB) strain, two fragments were amplified by PCR using *A. nidulans* ABPU1 genomic DNA as a template together with the primers listed in Table S1. The *pyrG*-*alcA*(p) cassette, which contains the *A. nidulans pyrG* marker and *alcA* promoter, was used as the PCR template with the primers CagsB-PU and CagsB-PL. The three fragments were used as substrates for the second round of PCR fusion. The resulting major PCR product was gel-purified and used to transform the ABPU1 strain (Figure S3).

To construct the disruption cassette for *agsA* in the CagsB strain, we used the same PCR fusion strategy. In the first round of PCR, the primers agsA-F and agsA-LL2 (for amplicon 1), agsA-RU2 and agsA-R (for amplicon 2), and argB-F and argB-R (for amplicon 3) were used (Table S1). The primers agsA-F and agsA-R were used for the second round of PCR. The resulting major PCR product was gel-purified and used to transform the ABPU1 strain.

### Construction of the Complementation Strain of the agsB Disruptant

To construct the complementation strain of the *agsB* disruptant, we amplified the full-length *agsB* gene, including its native promoter region (2 kb upstream of the 5′ end of the open reading frame [ORF]) and the terminator region (0.4 kb downstream of the 3′ end of the ORF), by means of PCR using the primers agsB-LU and agsB-T400R-HindIII (Table S1). The restriction enzyme sites for *Hin*dIII were found 1.4 kb upstream of the 5′ end of the *agsB* ORF and were amplified using the primer agsB-T400R-HindIII. The PCR fragment was digested with *Hin*dIII, and then introduced into the *Hin*dIII site of pUCpyroA (kindly provided by H. Horiuchi of the University of Tokyo), which contained the *A. nidulans pyroA* gene as the selectable marker. The resulting plasmid (pPAagsB) was used to transform the *agsB* disruptant strain.

### Quantitative RT- PCR

Real-time RT-PCR was performed using the Mini Opticon real-time PCR system (Bio-Rad Laboratories, Hercules, CA, USA) with SYBR Green detection, according to the manufacturer’s instructions. For reaction mixture preparation, the DyNAmo SYBR Green qPCR kit (Finnzymes Oy, Espoo, Finland) was used. We used the primer sets described previously [10] and listed in Table S1 for quantifying cell wall–related gene expression. An equivalent amount of cDNA, obtained from reverse transcription reactions using an equivalent amount of total RNA, was applied to each reaction mixture. The histone H2B gene was used as a normalization reference (an internal control) for target gene expression ratios. Statistical analyses were based on Welch’s *t*-test, and significance was defined as a *P*-value of <0.05.

### Growth Inhibition Testing of Mutants

We evaluated the sensitivity of the strains to the cell wall stress–inducing compounds micafungin, CFW, and Congo Red (CR) by plotting the dose response for colony growth using the plate dilution method. The chemicals were added from 1000-fold concentrated stock solutions in water (micafungin and CR) or in 0.1 M NaOH (CFW). Conidial suspensions of each mutant (a total of 1×10^3^ cells) were spotted on the centers of CD plates containing various concentrations of micafungin (0.001 and 0.01 µg/mL), CFW (5 and 10 µg/mL), and CR (1, 5, 20, 40, and 80 µg/mL). The dose response was determined 4 days after incubation at 37°C by plotting the mean diameters of the colonies on the treated media as a percentage of those on the control medium. Each experiment was performed in triplicate.

### Assay for Adsorption of CR to Hyphae

Mycelia cultured in CD medium for 24 h (500 mg fresh weight) were suspended in 50 mL of CR solution (40 µg/mL), and the solutions were rotated for 1 h at room temperature. After the reaction, the mycelia were removed by filtration through a 70 µm cell strainer (BD Biosciences, San Jose, CA, USA), and the absorbance of the supernatants was measured (UVmini-1240, Shimazu Co., Ltd., Tokyo, Japan) at 500 nm; in this assay, high absorbance indicates that most of the CR remained in the supernatant instead of being adsorbed to the mycelia. Each experiment was performed in triplicate. Statistical analyses were based on Welch’s *t*-test, and significance was defined as a *P*-value of <0.05.

### Assay for Cell Wall Susceptibility to Lysing Enzymes

Susceptibility of the fungal cell wall to Lysing Enzymes, a commercial preparation containing β-1,3-glucanase and chitinase (Sigma, St. Louis, MO, USA), was assayed according to the modified methods described in Fujikawa et al. [21]. Washed 1-d-old mycelia (30 mg fresh weight) grown in CD or CDTF medium at 37°C were resuspended in 1 mL of 0.8 M NaCl in sodium phosphate buffer (10 mM, pH 6.0) containing 10 mg/mL Lysing Enzymes. To determine the effect of additional α-1,3-glucanase on lysis of the fungal cell wall by Lysing Enzymes, 20 µg of purified α-1,3-glucanase from *Bacillus circulans* KA-304 [25] was added to the enzyme–buffer mixture. After 1, 2, and 4 h of incubation at 30°C, the number of protoplasts generated from the mycelia was counted using a hemocytometer (A106, SLGC, Tokyo, Japan). Mycelia incubated in 0.8 M NaCl in sodium phosphate buffer (10 mM, pH 6.0) containing no enzyme were used as controls.

### Fractionation of Cell Walls by Alkali Treatment

The steps used for cell wall fractionation are summarized in Figure S4. Mycelia cultivated in liquid medium were collected by filtration, freeze-dried, and pulverized in liquid nitrogen, and then 2 g of the mycelial powder was suspended in 80 mL of 0.1 M phosphate buffer (pH 7.0). The mycelial suspension was autoclaved at 120°C for 60 min and centrifuged at 10,000×*g* for 15 min. The supernatant was retained, and the pellet was resuspended in 80 mL of 0.1 M phosphate buffer (pH 7.0), autoclaved, and centrifuged again at 10,000×*g* for 15 min. The supernatants from the first two centrifugations were combined, dialyzed against water, and lyophilized, resulting in the hot-water-soluble (HW) fraction. The pellet was treated with 2 M NaOH for 24 h at 4°C. After centrifugation of the reaction products (10,000×*g*, 15 min), the supernatant was designated as the alkali-soluble fraction (AS) and the pellet was designated as the alkali-insoluble fraction (AI). The alkali-soluble fraction was neutralized with acetic acid (17 M), dialyzed against water, and centrifuged (10,000×*g*, 15 min). The supernatant was designated AS1, and the precipitate as AS2. The AI fraction was suspended in water, neutralized (as above), and dialyzed against water. All fractions were freeze-dried and the weight of each fraction was measured.

### Quantitative Determination of the Carbohydrate Composition of the Cell Wall Fractions

Each freeze-dried cell wall fraction (10 mg dry weight) was incubated in 0.2 mL of 12.5 M H_2_SO_4_ at 4°C for 16 h. Distilled water (4.8 mL) was added to the cell wall solution, resulting in a final concentration of 0.5 M H_2_SO_4_. After the cell wall was hydrolyzed at 100°C for 12 h, the hydrolysate was neutralized with barium carbonate and centrifuged to remove the resulting barium sulfate. The carbohydrate composition of the supernatant was determined using high-performance anion-exchange chromatography (HPAC) with a pulsed electrochemical detector and an anion-exchange column (Carbo PAC PA-1, 4×25 mm, Dionex, Sunnyvale, CA, USA) at a flow rate of 1 mL/min for 16 min. Isocratic elution was performed with 18 mM NaOH. The column was stabilized for 20 min before injection. To quantify the monosaccharides, we used glucose (Wako Pure Chemical Industries, Ltd., Osaka, Japan), mannose (Wako), galactose (Wako), glucosamine hydrochloride (Sigma), and galactosamine hydrochloride (Sigma) as standards.

### ^13^C-NMR Analysis of Cell Wall Fractions

The bacterial polysaccharide mutan, which was enzymatically synthesized by *GTF1* of *Streptococcus mutans* and mainly composed of α-1,3-glucan [S. Yano et al., unpublished data], and the AS2 fractions of the control (wild-type) and *agsA* mutant strains (each 2 mg) were suspended in 540 µL of D_2_O and autoclaved at 120°C for 60 min. Then, 60 µL of 5 M NaOH was added to each suspension, and the fractions were sonicated and dissolved. One drop of Me_2_SO-d_6_ (deuterated dimethyl sulfoxide, DMSO-d_6_) was added to each solution, and the solutions were centrifuged (3000×*g*, 5 min). ^13^C-NMR spectra of the supernatants were obtained using a JEOL (Tokyo, Japan) JNM-ECX400P spectrometer at 400 MHz, at 35°C for 60 h, with an external standard (tetramethylsilane).

### Treatment of the AS2 and AI Fractions with α-1,3-glucanase or β-1,3-glucanase

The AS2 and AI fractions (5 mg pure powder) from the control (CNT), *agsB* disruption, and CagsB strains were dissolved in 1 mL of potassium phosphate buffer (50 mM, pH 6.5) containing 10 µg of purified α-1,3-glucanase from *Bacillus circulans* KA-304 [25] or in 1 mL of acetate buffer (50 mM, pH 5.0) containing 500 µg of purified β-1,3-glucanase from *A. niger* (Sigma). The reaction was performed at 37°C for 12 h (α-1,3-glucanase) or at 55°C for 12 h (β-1,3-glucanase). After the reaction, the samples were centrifuged at 10,000×*g* for 10 min. The supernatants were retained, and each pellet was resuspended in the same buffer and enzyme mixture as before, incubated under the same conditions, and centrifuged as described above. The supernatants were removed and the procedure was performed a third time. The supernatants from the three centrifugations were combined and hydrolyzed with 2 M H_2_SO_4_. The liberated saccharides were quantified by means of HPAC with a pulsed electrochemical detector and an anion exchange column (Carbo PAC PA-1, 4×25 mm, Dionex) using glucose as the standard.

### Linkage Analysis of Glucan

Pachyman, which is a β-1,3-glucan, the bacterial mutan, and the alkali-soluble (AS2) fraction of the control strain were methylated using the modified method of Ciucanu and Kerek [26]. Each fraction (0.5 mg) was suspended in NaOH/dimethyl sulfoxide (DMSO) solution (10 mg NaOH in 0.5 mL of DMSO), and 0.2 mL of methyl iodide was added. The mixture was stirred for 15 min in a closed vial at room temperature. Water (2 mL) and chloroform (0.5 mL) were then added, the contents of the vial were mixed, and the aqueous phase was removed. The chloroform layer was mixed again with 2 mL water, and the aqueous phase was removed. Then, 1 mL of methylbenzene was added to the chloroform phase and the mixture was dried down. The dried material was resuspended in 0.5 mL of the NaOH/DMSO solution, and the methylation procedure was repeated.

Next, 0.5 mL of 2 M trifluoroacetic acid was added to the dried material (the methylated sample), which was hydrolyzed at 90°C for 1 h. Subsequently, 1 mL of methylbenzene was added to the sample and evaporated to dryness. The hydrolyzed sample was mixed with 0.5 mL of 0.25 M NaBH_4_ and reduced by incubation for 16 h at room temperature. Acetic acid and methylbenzene (approximately 0.4 mL and 1 mL, respectively) were added to the reduced sample and the mixture was evaporated to dryness. Methanol (1 mL) was added to the dried material and the mixture was evaporated to dryness; this step was performed three times. Pyridine (0.2 mL) and acetic anhydride (0.2 mL) were added to the sample, which was then acetylated by heating at 90°C for 20 min. Subsequently, 1 mL of methylbenzene was added to the acetylated sample and evaporated to dryness. The sample was mixed with chloroform (0.5 mL) and water (2 mL×3 times); each time, the aqueous phase was removed. Then, 1 mL of methylbenzene was added to the final chloroform layer and evaporated to dryness, and the sample was resuspended in 50 mL of chloroform.

The methylated sugars were analyzed using a gas chromatography system (GC-2010/GCMS-QP2010; Shimazu) with an HP-5 column (Agilent Technologies, Inc., Santa Clara, CA, USA). The identification of the peaks was confirmed by comparison with information in the Shimazu mass spectral libraries.

### Freeze-Substitution Electron Microscopy

Cells were collected from liquid media and snap-frozen by plunging them into a melting propane–isopentane mixture cooled with liquid nitrogen [27]. Specimens were then freeze-substituted in acetone containing 2% osmium tetroxide at –80°C, and embedded in epoxy resin. Ultrathin sections were cut to a thickness of 70 to 80 nm, stained with uranyl acetate and lead citrate [28], covered with Super support film (Nisshin EM, Tokyo, Japan), and examined using a JEM-1200EX transmission electron microscope (JEOL) at 80 kV.

## Results

### Isolation and Characterization of an A. nidulans agsA Disruptant (DagsA)

To investigate the role of the α-1,3-glucan synthase genes in *A. nidulans*, we first generated DagsA mutants with a disruption of the *agsA* gene. The *agsA* gene of the parental strain was disrupted using the targeted replacement method by selecting double-crossover events from homologous recombination. We constructed the disruption cassettes, which replaced the region between part of the promoter region of *agsA* and part of the coding region (including the start codon) of the *A. oryzae pyrG* gene as the selectable marker in *A. nidulans*, by means of PCR fusion and introduced the cassette into the parental strain (Figure S1). Correct integration of the cassettes and gene disruption were confirmed by means of PCR and Southern blot analysis (Figure S1). The results of quantitative RT-PCR revealed that the level of transcription of the *agsA* gene in the control strain was very low (Figure S5A and described below). Moreover, the transcripts of the *agsA* gene were scarcely detected in the *agsA* disruption strain (Figure S5A). The hyphal morphology, conidiation, phialide formation, and colony diameter of the *agsA* disruptants on CD plates were similar to those of the control strain (Figure S5B, S5C; data not shown). No significant differences in sensitivities to the cell wall stress–inducing compounds micafungin, CFW, and CR were observed between the *agsA* disruption strain and the control strain (Figure S5D). The susceptibility of the mycelia of the *agsA* disruption strain to Lysing Enzymes, a mixture that contains β-1,3-glucanase and chitinase, was not significantly different from that of the control strain (Figure S5E). In addition, the transcription levels of cell wall–related genes, such as the genes encoding β-glucan synthase and chitin synthase (data not shown), and the cell wall components that we examined were not altered in the *agsA* disruption strain compared with the control strain (described below). These results suggested that AgsA is not responsible for the biosynthesis of cell wall α-1,3-glucan under normal growth conditions.

### Isolation of the agsB Disruption Strains, agsA agsB Double-disruption Strains, Conditional-agsB Strains, and Conditional-agsB with agsA Disruption Strains of A. nidulans

To analyze the functions of the *agsB* gene in *A. nidulans*, we constructed two types of strains: strains with disruption of the *agsB* gene (DagsB strains), and conditional-*agsB* (CagsB) strains in which expression of *agsB* was regulated by the *A. nidulans alcA* promoter. We isolated the *agsB* disruption strains by disrupting the *agsB* gene of the parental strain using the targeted replacement method. The disruption cassette, which replaced an *agsB* region containing part of the promoter region and part of the coding region (including the start codon) with the *A. oryzae argB* gene as the selectable marker, were constructed by means of PCR fusion and introduced into the control strain (Figure S2). Correct integration of the disruption cassette and successful gene disruption were confirmed by means of PCR and Southern blot analysis (Figure S2). To confirm whether the *agsA* gene could compensate for the disruption of *agsB*, we generated an *agsA agsB* double-disruption strain (DagsA-DagsB). The *agsB* gene of the *agsA* disruption strain was also disrupted using the targeted replacement method. Correct integration of the cassettes and gene disruption were confirmed by means of PCR and Southern blot analysis (data not shown).

Next, we isolated several CagsB strains. We constructed a replacement cassette that replaced the native *agsB* promoter (–1000 bp from the start codon) with the *alcA* promoter and that also contained the *pyrG* selectable marker, and introduced the cassette into the parental strain (Figure S3). The genomic structure of the resulting CagsB strains was confirmed by means of PCR and Southern blot analysis (Figure S3). Three independent mutant lines were established to provide replication in the experiments. To confirm the regulation of *agsB* expression in the CagsB strains, we performed transcriptional analysis by means of quantitative RT-PCR under several medium conditions. The *alcA* promoter has been reported to activate gene expression in the presence of carbon sources such as ethanol, glycerol, and threonine, and to repress it in the presence of glucose [29]. First, we tested the CagsB strains on CD (1% glucose) medium as the *agsB*-repressing conditions. After 24 h of culture in CD liquid medium, the CagsB strains exhibited significantly lower *agsB* expression than the control strain (Figure S6A, left). When the CagsB strains were cultured in CDTF medium (0.1 M threonine, 0.1% fructose to replace the glucose), *agsB* expression in the CagsB strains was considerably (and significantly) higher than that of the control strain (Figure S6A, right). These results showed that the *alcA* promoter in the CagsB strains successfully regulated *agsB* expression in response to differences in the carbon source in the medium. We also generated a CagsB strain with an *agsA* disruption (CagsB-DagsA). We constructed a disruption cassette that replaced an *agsA* region that included part of the promoter region and part of the coding region (including the start codon) by the *A. oryzae argB* gene as the selectable marker by means of PCR fusion and introduced the cassette into the CagsB strain. Correct integration of the cassette and gene disruption were confirmed by means of PCR and Southern blot analysis (data not shown).

### The Growth Characteristics of the agsB Mutants

**Figure 1:**
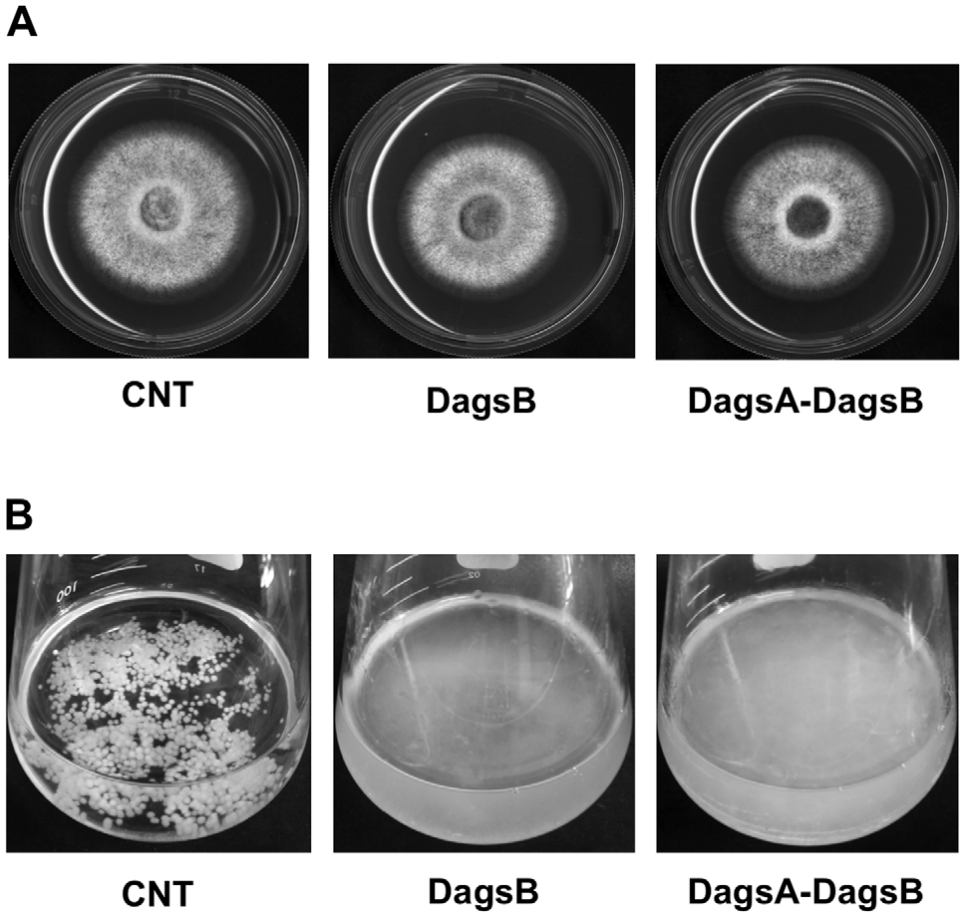
Phenotypes of the *agsB* disruption and *agsA agsB* double-disruption strains. (**A**) Mycelial growth of the control (CNT), *agsB* disruption (DagsB), and double-disruption (DagsA-DagsB) strains cultured on solid CD medium for 4 d. (**B**) Growth characteristics of the control (CNT), *agsB* disruption (DagsB), and double-disruption (DagsA-DagsB) strains. Conidia (final concentration, 5×10^5^/mL) of each strain were inoculated into CD liquid medium and rotated at 160 rpm at 37°C for 18 h.

**Figure 2:**
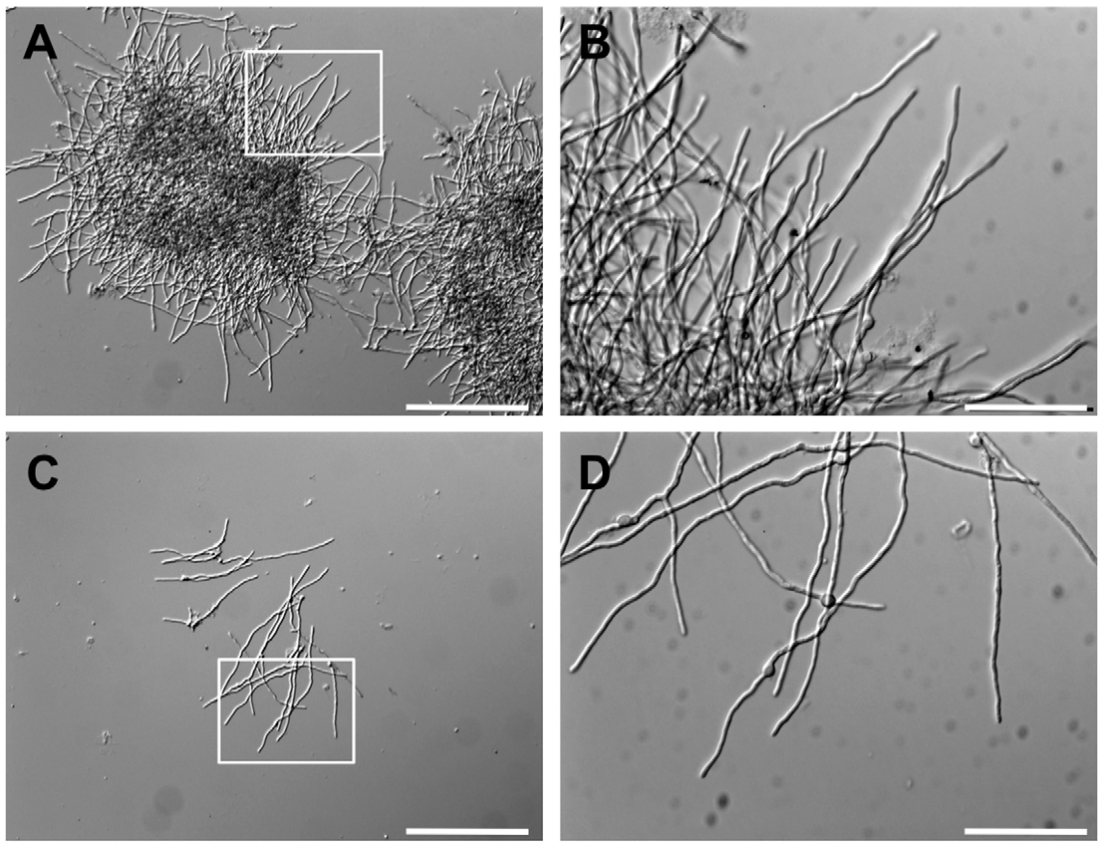
Hyphal morphology of the control and *agsB* disruption strains grown in CD liquid medium for 12 h at 37°C. (**A**) Hyphal pellets of the control strain, showing hyphal aggregation (dark areas). Scale bar = 200 µm. (**B**) Hyphal tip morphology of the control strain. High magnification of the hyphal image in the white frame in panel A. Scale bar = 50 µm. (**C**) Dispersed hyphae of the *agsB* disruption strain (DagsB). Scale bar = 200 µm. (**D**) Hyphal tip morphology of the DagsB strain. High magnification of the hyphal image in the white frame in panel A. Scale bar = 50 µm.

To understand the role of *agsB*, we examined the growth characteristics of the *agsB* mutant strains. The colonial growth rate and conidiation of the *agsB* disruption (DagsB) and double-disruption (DagsA-DagsB) strains on CD plates were similar to those of the control strain (Figure 1A; data not shown). In contrast to the results obtained with plate media, the growth characteristics of the *agsB* disruption and control strains were easily distinguishable in liquid culture (Figure 1B). The hyphae of the *agsB* disruption strain were obviously dispersed in liquid CD medium, whereas the control strain formed hyphal pellets (Figure 1B). The control strain showed an aggregation of hyphae in liquid culture (Figure 2, dark area), whereas the hyphae of the *agsB* disruption strain were scarcely aggregated (Figure 2). In contrast to the differences in the growth characteristics, the microscopic hyphal tip morphology and the phialide formation of the *agsB* disruption strain were similar to those of the control strain (Figure 2; data not shown). Also, the hyphae of the double-disruption strain were dispersed in liquid CD medium (Figure 1B).

To further confirm whether the phenotypic alterations in the *agsB* disruption strain were caused by disruption of the *agsB* gene, the growth characteristics of the CagsB strains were observed under *agsB-*repressing and -inducing conditions. No differences in colonial growth were observed between the CagsB and control strains on either CD (repressing conditions) or CDTF (inducing conditions) plates (Figure S6B). Conidiation on CD plates was not significantly different between the control and CagsB strains (Figure S6C). In liquid CD medium (*agsB*-repressing conditions), the hyphae of the CagsB strain were dispersed (Figure S6D, left), as in the case of liquid culture of the *agsB* disruption strains (Figure 1B). Under *agsB*-inducing conditions (liquid CDTF medium), both the CagsB and control strains formed hyphal pellets resembling those produced by the control strain cultured in liquid CD medium (Figure S6D, right). In addition, complementation of the *agsB* disruptants with the authentic *agsB* gene, including its native promoter, rescued the *agsB* disruptant and restored the wild-type phenotype (data not shown). These data indicated that the dispersed growth characteristic observed in the *agsB* disruption mutants was caused by disruption of the *agsB* gene.

The freeze-substitution electron microscopy assay revealed that the ultrastructures of the CagsB strains under *agsB*-repressing and *agsB*-inducing conditions were similar to those of the control strain (Figure S7). No significant differences in the thickness of the cell wall were observed between the control and CagsB strains (Table S2). In the CagsB with *agsA* disruptant strain (CagsB-DagsA), no obvious phenotypic alterations were observed compared with CagsB. The colonial growth rate and conidiation were indistinguishable between the CagsB-DagsA strain and the parental CagsB strain on both CD (*agsB*-repressing conditions) and CDTF (*agsB*-inducing conditions) plates (data not shown). The hyphae of the CagsB-DagsA strain were dispersed in liquid CD medium (*agsB*-repressing conditions in the CagsB-DagsA strain), similar to those seen in the parental CagsB, *agsB* disruption, and double-disruption strains (data not shown). These observations suggested that the *agsA* gene did not compensate for disruption or repression of *agsB* under our experimental conditions.

### Expression Profiles of Genes Involved in Cell Wall Biosynthesis

**Figure 3:**
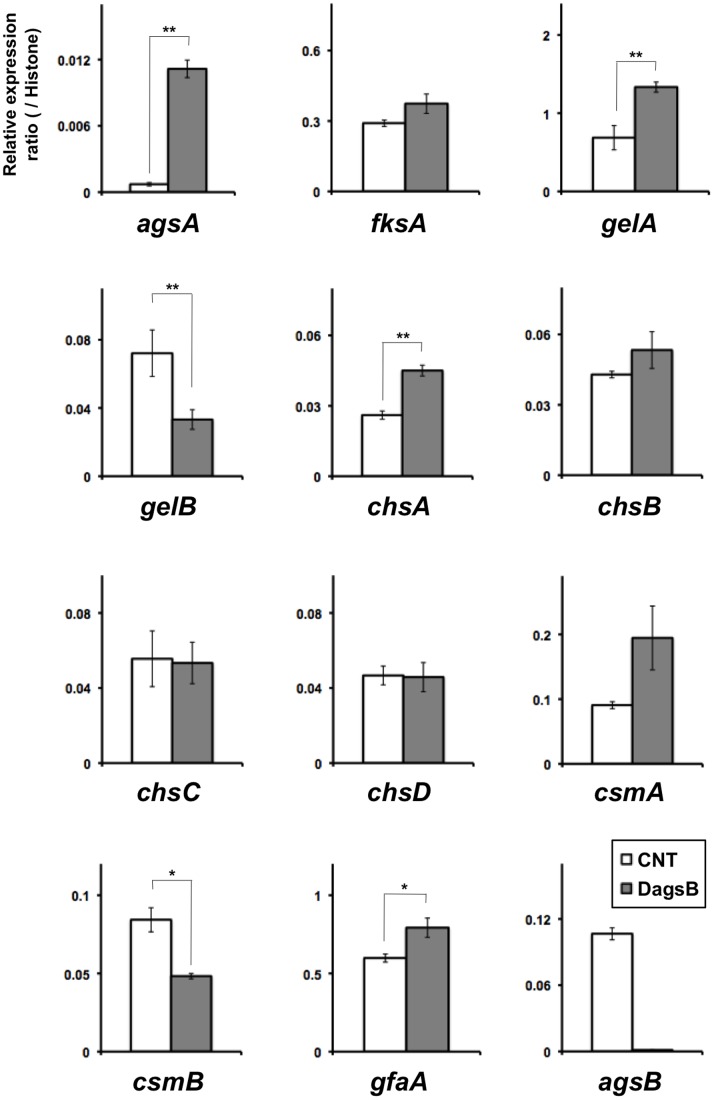
Expression of cell wall–related genes in the control (CNT) and *agsB* disruption (DagsB) strains. Strains were grown in CD liquid medium at 37°C. Levels of transcription of the indicated genes were determined by means of quantitative RT-PCR of total RNA using the gene-specific primers reported previously by Fujioka et al. [10] (Table S1). Each value represents the ratio of expression relative to the histone H2B gene in each strain. Error bars represent the standard error of the mean calculated for three replicates (*P<0.05, **P<0.01).

**Figure 4:**
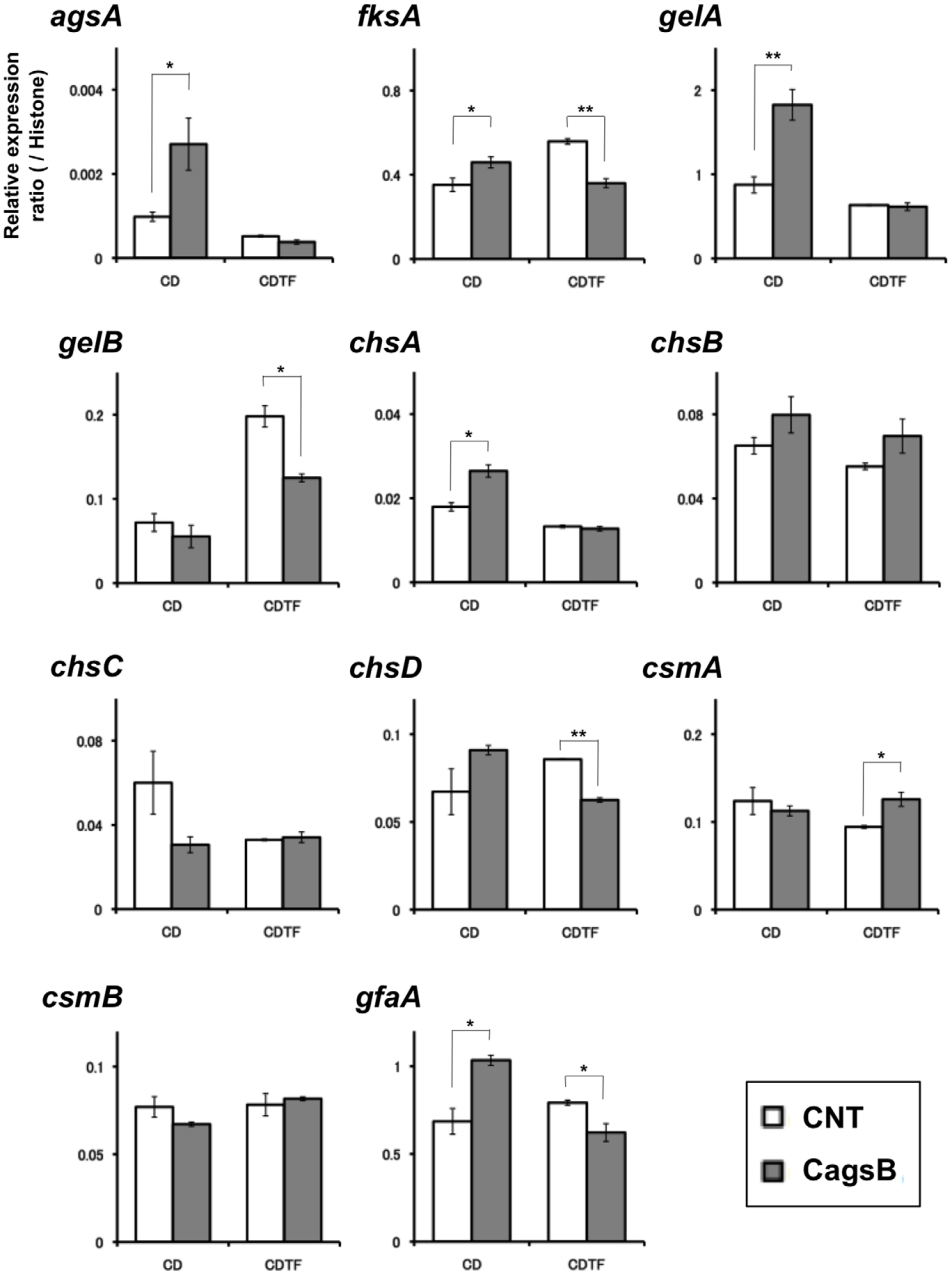
Expression of cell wall–related genes in the control (CNT) and CagsB strains. Strains were grown in CD liquid medium (*agsB*-repressing conditions) or CDTF liquid medium (*agsB*-inducing conditions) at 37°C. Levels of transcription of the indicated genes were determined by means of quantitative RT-PCR of total RNA using the gene-specific primers reported previously by Fujioka et al. [10] (Table S1). Each value represents the ratio of expression relative to the histone H2B gene in each strain. Bars represent the standard error of the mean calculated for at least three replicates (*P<0.05, **P<0.01).

To test whether the expression of genes associated with cell wall biogenesis was affected by the disruption of *agsB* or by the level of *agsB* expression, we analyzed the levels of transcription of several cell wall–related genes. We analyzed the transcriptional levels of *agsA* and *agsB* (α-1,3-glucan synthase genes); *fksA* (a β-1,3-glucan synthase gene); *gelA* and *gelB* (β-1,3-glucanosyl transferase genes); *chsA*, *chsB*, *chsC*, *chsD*, *csmA*, and *csmB* (chitin synthase genes); and *gfaA* (a glutamine-fructose-6-phosphate amidotransferase gene) by means of quantitative RT-PCR in the *agsB* disruption strain (DagsB; Figure 3) or in the CagsB strain (Figure 4) after treatment with *agsB*-repressing and inducing conditions. In the *agsB* disruption strain, in which *agsB* expression was scarcely detected (Figure 3), the expression of *agsA* increased significantly, although the basal level of *agsA* expression was still less than 1% of that of *agsB* in the control (Figures 3, S5). In addition, levels of transcription of several cell wall–related genes (*gelA*, *chsA*, and *gfaA*) were significantly up-regulated in the *agsB* disruption strain (Figure 3); however, transcription of *gelB* and *csmB* were significantly down-regulated in the disruptant. In the CagsB strain, the expression of *agsA*, *fksA*, *gelA*, *chsA*, and *gfaA* were significantly up-regulated under the *agsB*-repressing conditions (CD medium; Figure 4), as was the case in the *agsB* disruption strain for all except *fksA*. In contrast, under the *agsB*-inducing conditions (CDTF medium), the expression levels of *fksA*, *gelB*, *chsD*, and *gfaA* were significantly reduced in the CagsB strain (Figure 4); however, *csmA* was significantly up-regulated under these conditions. These results suggested that alteration of the α-1,3-glucan amount was counterbalanced by alterations in the amount of another cell wall component such as β-1,3-glucan or chitin, so we examined this possibility.

### Susceptibility to Cell Wall–Stress Compounds and Cell Wall–Degrading Enzymes in the agsB Mutants

**Figure 5:**
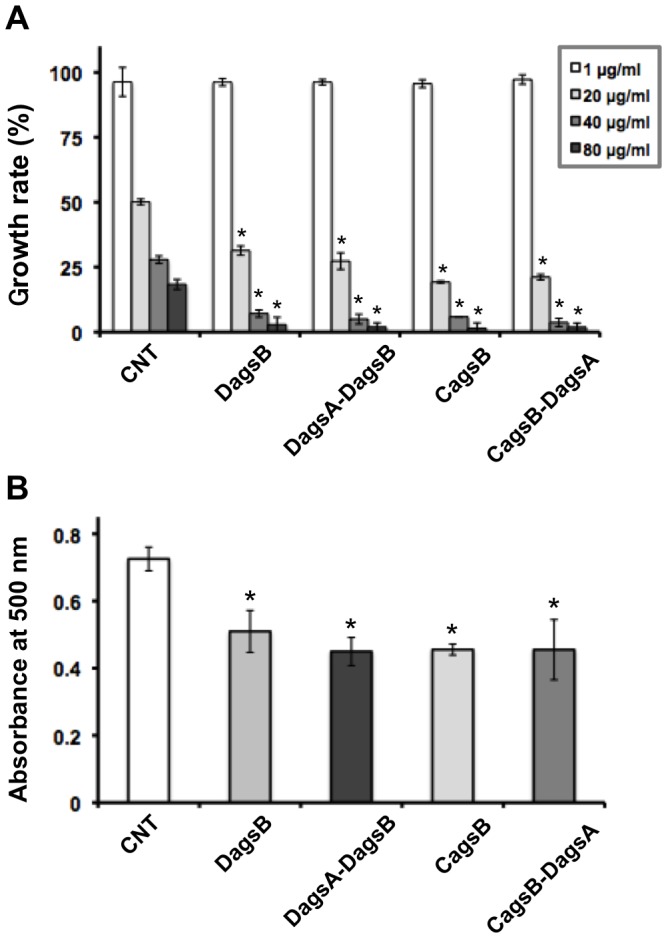
Sensitivity of the control (CNT), *agsB* disruption (DagsB), *agsA agsB* double disruption (DagsA-DagsB), CagsB, and CagsB with *agsA* disruption (CagsB-DagsA) strains to Congo Red (CR). (**A**) Growth rate after 4 days of growth on CD medium at the indicated concentration of added CR. Error bars represent the standard deviations (*n* = 3). *, significantly different from the control (*P*<0.01). (**B**) Adsorption of CR to the hyphae of each strain. Mycelia cultured in CD for 24 h (500 mg fresh weight) were suspended in 50 mL of CR solution (40 µg/mL) and rotated (160 rpm) for 1 h at room temperature. After the reaction, the mycelia were removed by filtration, and the absorbance of the supernatant was measured at 500 nm. Absorbance levels indicate the levels of CR remaining in the supernatants (i.e., not adsorbed to the hyphae). Error bars represent the standard error of the mean calculated for three replicates (*P<0.05).

To investigate the consequences of cell wall alteration caused by the disruption of *agsB* or by the control of *agsB* gene expression, we first tested the sensitivities of the mutant strains to cell wall stress–inducing compounds. The *agsB* disruption strain showed greater sensitivity than the control to CR, which interferes with cell wall assembly (Figure 5A). Under the *agsB*-repressing conditions, the CagsB strain also showed increased sensitivity to CR (Figure 5A). The DagsB and CagsB strains were barely able to grow on CD plates containing 80 µg/mL of CR, whereas the growth rate of the control strain was still approximately 20% of normal on CD containing 80 µg/mL of CR (Figure 5A). The median effective concentrations (EC_50_) of CR for the control, DagsB, and CagsB strains were 18.1, 11.8, and 8.9 µg/mL, respectively. No significant differences in the sensitivity to CR were observed in the DagsA-DagsB double-disruption strain compared with the DagsB strain or the CagsB-DagsA strain compared with the CagsB strain (Figure 5A). On CDTF plate medium (*agsB-*inducing conditions for the CagsB strain), no obvious differences in the sensitivity to CR were detected between the control and the CagsB strain. The minimum inhibitory concentration (MIC) for the CagsB strain was the same level as for the control strain (MIC >100 µg/mL; data not shown). Next, we measured the adsorption of CR to the hyphae of the control, DagsB, DagsA-DagsB, CagsB, and CagsB-DagsA strains. The amount of CR adsorbed to the hyphae of these *agsB* mutants grown in liquid CD culture medium was significantly greater than that of the control strain (i.e., low absorbance by the supernatant; Figure 5B). In contrast to the inhibitory effect seen with CR, the DagsB and DagsA-DagsB strains were not sensitive to either micafungin (an inhibitor of β-glucan synthase) or CFW (a chitin binding agent) (data not shown). Similarly, the CagsB and CagsB-DagsA strain were not sensitive to micafungin or CFW under either *agsB-*repressing or -inducing conditions (data not shown).

**Figure 6:**
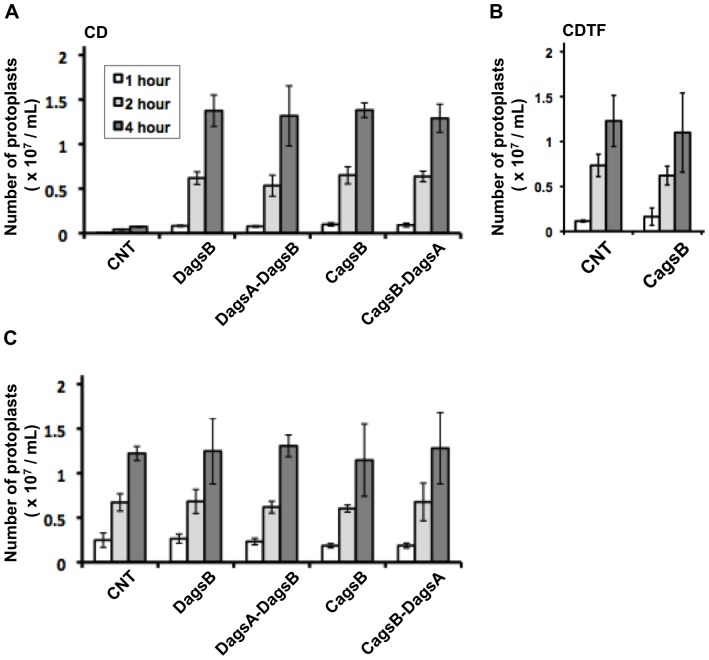
Cell wall susceptibility of the control (CNT), *agsB* disruption (DagsB), *agsA agsB* double disruption (DagsA-DagsB), CagsB, and CagsB with *agsA* disruption (CagsB-DagsA) strains to Lysing Enzymes. (**A**) Susceptibility of mycelia cultured in CD liquid medium (*agsB-*repressing conditions). Mycelia cultured in CD medium for 24 h (30 mg fresh weight) were digested in reaction buffer (10 mM phosphate buffer, pH 6.0) containing 10 mg/mL Lysing Enzymes. After 1, 2, and 4 h of incubation at 30°C, the number of protoplasts in each sample was determined using a hemocytometer. Error bars represent the standard deviations (*n* = 3). All values of the mutants differed significantly from that of the control strain. (**B**) Susceptibility of the control and CagsB strains cultured in CDTF liquid medium (*agsB-*inducing conditions). Mycelia cultured in CDTF medium for 24 h (30 mg fresh weight) were digested, and the number of protoplasts in each sample was determined according to the method described above. None of the differences were statistically significant. (**C**) Effect of combined treatment with α-1,3-glucanase and Lysing Enzymes on protoplast formation from fungal mycelia. Mycelia cultured in CD medium for 24 h were digested in reaction buffer containing both Lysing Enzymes and α-1,3-glucanase. The number of protoplasts generated in each sample was determined according to the method described above. None of the differences were statistically significant.

To examine whether *agsB* expression contributes to protection of the cell wall from cell wall–degrading enzymes, we tested the susceptibility of mycelia to Lysing Enzymes, which contains both β-1,3-glucanase and chitinase. For each strain, the number of protoplasts produced from the mycelia by lysis was measured after treatment with Lysing Enzymes. When mycelia cultured in CD liquid medium were treated with Lysing Enzymes, few protoplasts were generated from the control strain even after 4 h (Figure 6A); in contrast, large numbers of protoplasts were generated from the DagsB, DagsA-DagsB, CagsB, and CagsB-DagsA mycelia cultured in CD liquid medium (*agsB*-repressing conditions for the CagsB and CagsB-DagsA strains) after 4 h. On the other hand, when mycelia cultured in CDTF medium (*agsB*-inducing conditions for the CagsB and CagsB-DagsA strains) were incubated with Lysing Enzymes, the number of protoplasts generated from the CagsB strain was not significantly different from the number generated from the control strain (Figure 6B). The number of protoplasts may have increased for the CNT strain in CDTF, relative to the number in CD, as a result of the production of a weak cell wall in CDTF media. This weakness would result from changes in the alkali-insoluble cell wall fraction, which is composed of chitin and β-glucan (see below), which was reduced by up to 30% of the total cell wall fractions in the CNT strains cultured in CDTF media (data not shown). When mycelia were digested with both Lysing Enzymes and α-1,3-glucanase (Figure 6C), the numbers of protoplasts generated from the control strain increased significantly compared with those after treatment with the Lysing Enzymes alone, and the numbers of protoplasts generated during each incubation period were comparable to those generated from these *agsB* mutant strains (Figure 6B). These results suggested that the loss of α-1,3-glucan caused by the disruption or repression of *agsB* increased the sensitivity of the mycelial cell wall to Lysing Enzymes, suggesting the possibility that cell wall β-1,3-glucan and chitin are more accessible to β-1,3-glucanase and chitinase in mycelial cells with a decreased α-1,3-glucan content.

### Comparison of Cell Wall Components in the Control Strain and the ags Mutant Strains

**Figure 7:**
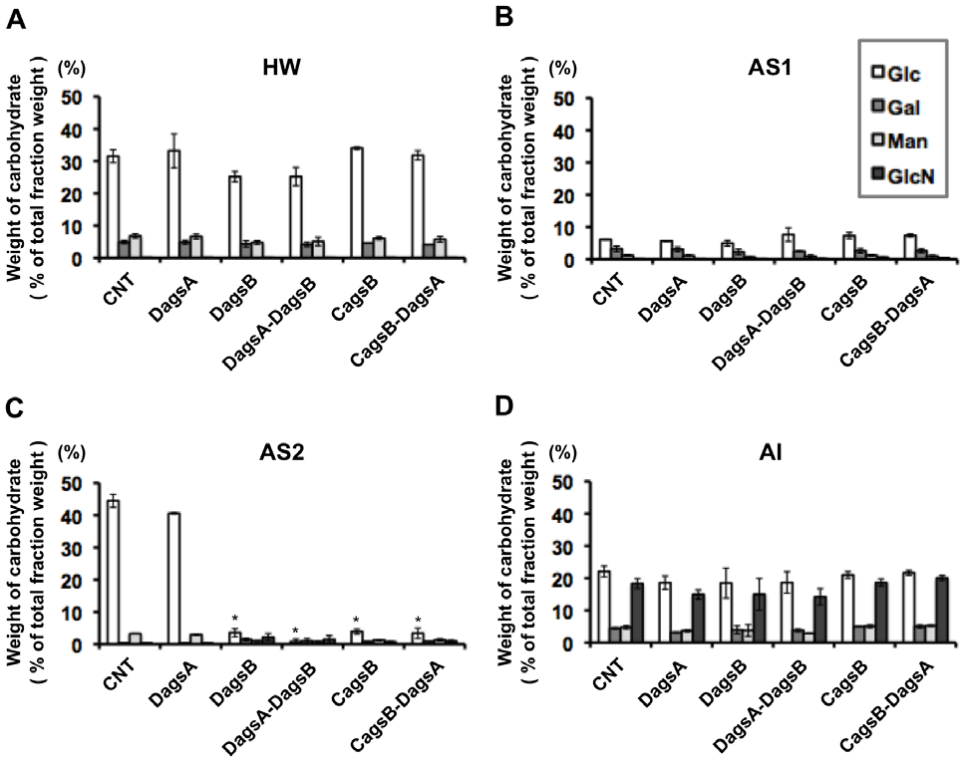
Cell wall composition of each cell wall fraction. The control (CNT), *agsA* disruption (DagsA), *agsB* disruption (DagsB), double-disruption (DagsA-DagsB), CagsB, and CagsB with *agsA* disruption (CagsB-DagsA) strains were cultured in CD liquid medium (*agsB*-repressing conditions for the CagsB and CagsB-DagsA strains). Monosaccharide compositions are reported for the (A) hot-water-soluble (HW), (B) alkali-soluble 1 (AS1), (C) alkali-soluble 2 (AS2), and (D) alkali-insoluble (AI) cell wall fractions (see Figure S4) as a percentage of the total dry weight of each fraction. Error bars represent the standard deviations calculated for at least three replicates. *, significantly different from the control (*P*<0.01). Glc, glucose; Gal, galactose; Man, mannose; GlcN, glucosamine.

Cell wall components of the control and *ags* mutant strains were fractionated based on their solubility in alkali ([30]; O. Mizutani et al., unpublished data). It has been reported that the alkali-soluble fraction in *A. fumigatus* is composed mainly of α-1,3-glucan with some galactomannan [31] and that the alkali-insoluble fraction is composed of chitin, β-1,6-branched β-1,3-glucan, and galactomannan [1], [30]. The cell wall components from the *A. nidulans* control and *ags* mutant strains were separated into four fractions (Figure S4), and the weight ratios of the fractions were measured (Table 1). The total recovery rate of the four fractions during the fractionation was 55% to 65% of the total cell dry weight in each strain. We determined the sugar compositions in the four fractions for each strain (Figure 7). The hot-water-soluble (HW) fraction was mainly composed of glucose, which accounted for 25 to 34% of the HW fraction (by weight) in each strain (Figure 7A). The AS1 fraction, which contained the water-soluble components after dialysis of the alkali-soluble fraction (Figure S4), was composed of glucose, galactose, and mannose (Figure 7B). However, these sugars together accounted for 8 to 11% of the total AS1 fraction weight in each strain. Glucose again dominated the sugars in the AS2 fraction, although it was only dramatically greater than the other sugars in the control and DagsA strains (Figure 7C). The alkali-insoluble (AI) fraction contained glucose, galactose, mannose, and glucosamine in both the control and *ags* mutant strains (Figure 7D). In each strain, the glucose and glucosamine contents of the AI fraction each accounted for about 20% of the AI fraction dry weight, and the galactose and mannose contents each accounted for about 5% of the fraction dry weight.

**Table 1:** Yield of each fraction obtained by alkali treatment of the control (CNT), *agsA* disruption (DagsA), *agsB* disruption (DagsB), double-disruption (DagsA-DagsB), conditional-*agsB* (CagsB), and CagsB with *agsA* disruption (CagsB-DagsA) strains.

	Yield^a^ (% of dry weight)^a^			
Strain	HW	AS1	AS2	AI
CNT	31.8	6.9	14.7	46.7
DagsA	31.7	5.7	14.4	48.2
DagsB	33.8	7.0	9.2	50.1
DagsA-DagsB	31.4	6.8	9.1	52.7
CagsB	36.9	10.3	7.1	45.7
CagsB-DagsA	40.3	7.0	6.6	46.2

a The yield of each fraction is expressed as the percentage of the total dry weight. Values represent the mean of triplicate measurements. Fractions: HW, hot-water-soluble; AS, alkali-soluble; AI, alkali-insoluble. Figure S4 shows the fractionation process.

No quantitatively significant differences were observed in the sugar compositions of the HW, AS1, and AI fractions between the control and *ags* mutant strains. On the other hand, statistically significant differences were visible in the sugar compositions in the alkali-soluble AS2 fraction, which was the precipitate that remained after dialysis of the alkali-soluble fraction, between the control and *agsB* mutant strains (Figure 7C). The AS2 fraction of the control and *agsA* disruption strains was mainly composed of glucose (i.e., the monosaccharide that makes up α-1,3-glucan), which represented 45% and 41% of the AS2 fraction, respectively. The results from the fractionation and the quantification of sugar contents revealed that the cell wall composition of the *agsA* disruption strain did not differ greatly from that of the control strain. Typical signals for α-1,3-glucan in ^13^C-NMR spectroscopic analysis were detected in the AS2 fraction of the control and *agsA* disruption strains (Figure S8; data not shown) [32]. The chemical shifts in the ^13^C-NMR corresponded to that of the bacterial mutan (Figure S8), which is composed primarily of α-1,3-glucan [S. Yano et al., unpublished data].

In addition, we performed a methylation (Me) analysis for the AS2 fraction of the control strain to determine the linkage structure of the glucans (Table S3). The presence of 2,4,6-tri-*O*-Me-Glc (1,3,5-triacetyl-2,4,6-tri-*O*-methyl-D-glucitol), which indicates 1,3-glucoside bonds, was mainly detected in the AS2 fraction of the control strain as in the case of the standard β-1,3-glucan pachyman and the bacterial mutan (Table S3). In contrast, we detected little 2,3,4,6-tetra-*O*-Me-Glc, 2,3,4-tri-*O*-Me-Glc, and 2,4-di-*O*-Me-Glc, which indicate (respectively) a non-reducing terminal, 1,6-glucoside bonds, and 3,6-branching points (Table S3). These data indicated that the AS2 fraction of the control strain contained mainly linear glucan consisting of 1,3-glucoside bonds. Taken together, these results from the ^13^C-NMR and the methylation analysis indicated that the AS2 fraction of the control strain was mainly composed of linear α-1,3-glucan.

In contrast, the glucose content of the AS2 fractions accounted for less than 5.0% by weight of the AS2 fraction in the DagsB, DagsA-DagsB, CagsB, and CagsB-DagsA strains when grown in CD liquid medium (*agsB*-repressing conditions for the CagsB and CagsB-DagsA strains) (Figure 7C). There was very little remaining glucan in the AS2 fraction derived from the DagsB and CagsB strains. Glucose was liberated from the AS2 fraction of these two strains by hydrolysis with β-1,3-glucanase but not with α-1,3-glucanase (Figure S9), indicating that the little glucan remaining in the AS2 fraction derived from the two mutants was β-1,3-glucan. These data suggested that the disruption of *agsB* and *agsB*-repressing conditions resulted in a marked decrease in the α-1,3-glucan content of the cell wall in the *agsB* disruption and CagsB strains. The cell wall components of the DagsA-DagsB and CagsB-DagsA strains were not distinguishable from those of the DagsB and parental CagsB strains, indicating that the *agsA* gene does not make a major contribution to α-1,3-glucan production (Table 1, Figure 7). The amount of the AS2 fraction and the remaining glucose content of the AS2 fraction of the DagsB strains were at the same low levels as those from the CagsB strains under the *agsB*-repressing conditions (Table 1, Figure 7C), suggesting that most of the cell wall α-1,3-glucan was lost in the CagsB strain.

## Discussion

*Aspergillus nidulans* possesses two α-1,3-glucan synthase genes, *agsA* and *agsB*. In the present study, we constructed disruption mutants of the *agsA* and *agsB* genes (DagsA and DagsB, respectively) and a conditional-*agsB* (CagsB) strain in which expression of *agsB* is regulated by the conditional promoter *alcA*. The disruption mutants of *agsA* did not show obvious phenotypic alterations under normal growth conditions. The hyphae of the DagsB strain were obviously dispersed in liquid CD medium (Figure 1B), and the hyphae of the DagsB strain were scarcely aggregated (Figure 2). Fontaine et al. [33] showed that latex beads coated with α-1,3-glucan bound to the swollen conidia of *A. fumigatus* and that α-1,3-glucan was solely responsible for conidial aggregation in *A. fumigatus*. Therefore, the dispersed hyphal growth of the *A. nidulans* DagsB strains appears to be attributable to the loss of α-1,3-glucan [33]. In the CagsB strain, *agsB* down-regulation also resulted in abnormal growth characteristics in liquid culture, whereas the strain showed normal growth on plate media regardless of the level of *agsB* expression. All phenotypes observed in the *agsB* disruption strains were consistent with those of the CagsB strain under the *agsB*-repressing conditions. In addition, the DagsA-DagsB and CagsB-DagsA strains showed no obvious phenotypic alteration compared with their respective parental strains (i.e., the DagsB and CagsB strains, respectively). Biochemical analysis of the cell wall polysaccharides revealed that both the disruption of *agsB* and the down-regulation of *agsB* expression in the CagsB strain led to almost complete loss of cell wall α-1,3-glucan. These results suggested that AgsB plays a crucial role in biosynthesis of cell wall α-1,3-glucan in *A. nidulans,* whereas AgsA plays only a small role.

Aspergilli generally possess several genes that encode an α-1,3-glucan synthase (AGS). *A. niger* is reported to have five AGS genes (*agsA* to *agsE*) [13], and *A. fumigatus* has three such genes (*AGS1*, *AGS2*, and *AGS3*) [11], [12]. *A. fumigatus AGS1* (*AfAGS1*) and *AGS2* (*AfAGS2*) are the counterparts of *A. nidulans agsB* and *agsA*, respectively. Disruption of *AfAGS1* led to a 50% decrease in the level of cell wall α-1,3-glucan, whereas deletion of *AfAGS2* had no detectable effect on glucan levels [11]. Deletion of either *AfAGS1* or *AfAGS2* yielded cells with altered hyphal morphology, altered phialide formation, and reduced conidiation [11]. During the preparation of this manuscript, a new paper appeared [14] in which a triple α-1,3-glucan synthase mutant of *A. fumigatus* was generated by deletions of the three α-1,3-glucan synthase genes, *AfAGS1*, *AfAGS2*, and *AfAGS3.* The triple AGS mutant of *A. fumigatus* was totally deficient in α-1,3-glucans, whereas the growth and conidial germination of the triple mutant were similar to those of the parental strain [14], and the conidiation of the triple mutant was slightly decreased compared to the parental strain, as was found for the single *AfAGS1* and *AfAGS2* mutants [11], [14]. In the present study, *A. nidulans agsA* disruptants did not show obviously altered phenotypes under normal growth conditions, and the α-1,3-glucan contents (i.e., the AS2 fraction) were the same as those of the control strain (Figure 7C). Although the colonial growth, conidiation, hyphal tip morphology, and formation of phialides were normal in the DagsB and CagsB strains, the disruption or down-regulation of *agsB* resulted in abnormal growth characteristics in liquid culture. The results of cell wall fractionation, determination of sugar compositions of the cell wall fractions, ^13^C-NMR analysis, and methylation analysis indicated that the AS2 fractions of the DagsB and CagsB strains contained very little α-1,3-glucan (Figures 7, S9; Tables 1, S3). Because the nearly complete loss of cell wall α-1,3-glucan caused by the disruption or down-regulation of *agsB* was greater than the 50% loss of cell wall α-1,3-glucan in the *AfAGS1* deletion strain [11], the contribution of *agsB* to α-1,3-glucan biosynthesis in *A. nidulans* seems to be larger than that of *AfAGS1* in *A. fumigatus*. These observations suggest that the *agsB* class of genes is important for the biosynthesis of α-1,3-glucan in both *A. nidulans* and *A. fumigatus*, but that the individual *agsB-*class genes seem to have marked differences in function.

Disruption of the third *A. fumigatus* AGS gene, *AfAGS3*, which is absent in *A. nidulans*, results in overexpression of *AfAGS1*, which may compensate for the loss of *AfAGS3* to maintain normal cell wall composition [12]. In addition, the lack of cell wall α-1,3-glucan in the *A. fumigatus* triple AGS mutant was accompanied by an increase in the β-1,3-glucan and chitin content of the mycelia [14]. In *A. nidulans*, the disruption and down-regulation of *agsB* led to a slight increase in *agsA* expression, but the expression level of *agsA* was significantly lower than that of *agsB* in the control strain (Figures 3, 4, S5). In addition, the phenotypes of the DagsA-DagsB and CagsB-DagsA strains did not differ from those of their parental strains (DagsB and CagsB, respectively), and the amount of each cell wall fraction derived from the DagsA-DagsB and CagsB-DagsA strains was the same as that of the respective parental strains (Table 1, Figure 7). These results suggested that *agsA* did not compensate for the loss of *agsB* expression in *A. nidulans* under our experimental conditions.

Our analysis of the transcriptional responses of the cell wall–related genes revealed that genes encoding β-1,3-glucan synthase and chitin synthases showed significantly increased expression levels in the DagsB strain and in the CagsB strain under *agsB*-repressing conditions (Figures 3, 4), suggesting that the loss of α-1,3-glucan was counterbalanced by alterations in the amount of another cell wall component such as β-1,3-glucan or chitin. In other fungi, compensatory changes in cell wall components have been reported. For example, the human pathogen *C. neoformans* possesses only one AGS gene (*AGS1*) involved in α-1,3-glucan synthesis [15]. A strain of *C. neoformans* in which the *AGS1* gene is disrupted shows a slow growth rate and a temperature-sensitive phenotype, and disruption of the *AGS1* gene results in a lack of capsule formation, which is one of the most important virulence factors in this pathogen [15]. In the *AGS1-*deleted strain, the loss of α-1,3-glucan is accompanied by an increase in chitin and chitosan contents and the redistribution of β-1,3-glucan from the alkali-soluble glucan fraction to the alkali-insoluble glucan fraction [16]. In the fission yeast *Schizosaccharomyces pombe*, the α-1,3-glucan synthase gene *mok1* (synonym *ags1*) is essential for the synthesis of cell wall α-1,3-glucan [34], [35]. A strain containing a mutated *mok1* gene shows slightly decreased α-glucan levels, but β-glucan levels are slightly increased. In contrast to the observations in other fungi, our analysis of cell wall polysaccharides showed that the cell wall chitin and β-1,3-glucan contents were not markedly altered in the *ags* mutants (Figures 7, S9; Table 1). In addition, no significant differences in staining of the cell wall with CFW or in the sensitivity to CFW were observed among the control and *agsB* disruption strains (data not shown). Although the *agsB* mutants showed a loss of cell wall α-1,3-glucan and up-regulation of transcription levels of several cell wall–related genes, the transcription levels of some cell wall–related genes, such as *gelB* and *csmB*, was down-regulated in the DagsB strain (Figure 3). In addition, the transcription levels of *gelB*, *chsC*, and, *csmB* tended to be down-regulated in the CagsB strain under the *agsB*-repressing conditions (Figure 4). Although the contribution of *gelB*, *chsC* and, *csmB* to biosynthesis of cell wall β-1,3-glucan and chitin is unclear in this experimental conditions, the lack of compensatory changes in the cell wall components in the *agsB* mutants might have been caused by differences in the contribution of each cell wall–related gene to the biosynthesis of cell wall polysaccharides.

Moreover, no marked differences in the ultrastructure or thickness of the cell wall were detected between the control and CagsB strains under either *agsB-*inducing or *agsB-*repressing conditions (Figure S7, Table S2). These observations suggested that the proportions of β-1,3-glucan and chitin in the cell wall were regulated appropriately because of the importance of the cell wall for fungal viability, regardless of the presence of cell wall α-1,3-glucan in *A. nidulans*.

Cell wall stress–inducing compounds often induce the expression of cell wall–related genes [10], [13], [36], [37]. In *A. nidulans*, levels of *agsB* transcripts are transiently increased by micafungin treatment, whereas *agsA* transcripts are maintained at low levels even after micafungin treatment [10]. As in the case of *A. nidulans*, the expression of *A. niger agsA* (which is absent from *A. nidulans*) and *agsE* (an orthologue of *agsB* in *A. nidulans*) is induced in the presence of CFW, sodium dodecyl sulfate, and caspofungin [13]. In addition, a disruptant of *A. niger agsA* was more sensitive than its parental (non-disrupted) strain to CFW [13]. Based on these observations, we predicted that the perturbation of cell wall α-1,3-glucan synthesis would affect the sensitivity to such cell wall stress–inducing compounds. Unexpectedly, the *A. nidulans agsB* disruption and CagsB strains did not show increased sensitivity to CFW or micafungin under any experimental conditions in the present study. Our analysis of cell wall polysaccharides revealed that the contents of cell wall chitin and β-1,3-glucan were not markedly altered in the DagsB and CagsB strains. Thus, the sensitivity of *A. nidulans* to CFW or micafungin is not dependent on the level of cell wall α-1,3-glucan. In contrast, the *agsB* disruption strain and the CagsB strain under *agsB*-repressed conditions both showed increased sensitivity to CR (Figure 5A), and the amount of CR adsorption to the hyphae of the DagsB and CagsB strains was significantly greater than that of the control strain (Figure 5B). Both CR and CFW interact with various polysaccharides, although β-1,3-glucan shows a strong interaction with CR but a weak interaction with CFW [38]. Although the amount of cell wall α-1,3-glucan was significantly lower in the DagsB and CagsB strains than in the control strain, the content of the AI fraction was the same (Table 1, Figure 7C, 7D). When we examined the adsorption of CR to each cell wall component (α-1,3-glucan, β-1,3-glucan, or chitin), the amount of CR adsorbed to α-1,3-glucan was significantly less than the amount adsorbed to β-1,3-glucan or chitin (A. Inaba et al., unpublished data). It is reasonable to hypothesize that the loss of cell wall α-1,3-glucan in the DagsB and CagsB strains led to increased exposure of β-1,3-glucan on the cell surface and to the resulting increased sensitivity to CR.

The outermost layer of the cell wall in many fungi consists primarily of α-1,3-glucan [16], [20], [21], [39], [40]. In *C. neoformans*, *ags1*Δ cells show a disorganization of the cell wall that accompanies the loss of α-1,3-glucan, and the perturbed cell wall can no longer serve as an attachment site for capsule fibers [16]. Ultrastructural studies of the dimorphic pathogen *H. capsulatum* revealed that the outermost layer of the cell wall consists primarily of α-1,3-glucan [39]. Rappleye et al. [20] also showed that α-1,3-glucan formed the outermost surface of *H. capsulatum* cells, based on cytological observations by immunofluorescence and immunoelectron microscopy. Fujikawa et al. [21] demonstrated a dynamic change in the composition of fungal cell wall polysaccharides during infection by the plant pathogen *M. grisea*. In *M. grisea*, α-1,3-glucan accumulates on the surface of germ tubes during plant infection, and the accumulation is induced by a plant cue [21]. In the infectious hyphae, the α-1,3-glucan was localized more outwardly than the β-1,3-glucan, resulting in increased tolerance to cell wall–digesting enzymes [21]. Our results of the susceptibility test against digestive enzymes and the biochemical analysis of cell wall polysaccharides are in good agreement with these observations. In *A. nidulans*, the disruption or repression of *agsB* resulted in increased susceptibility to cell wall–digesting enzymes (Figure 6A), and cell wall α-1,3-glucan was barely detected in hyphae of the DagsB strain or the CagsB strain cultured under the *agsB*-repressing conditions (Figure 7). Therefore, α-1,3-glucan seems to be localized to the outermost layer of the cell wall in *A. nidulans*.

In conclusion, our functional analysis of the *agsA* and *agsB* genes demonstrated that the two AGS genes were dispensable in *A. nidulans*; however, *agsB* was involved in determining the growth characteristics in liquid culture, although the colonial growth, conidiation, hyphal tip morphology, and formation of phialides were normal in the *ags* mutants. We were able to control the cell wall α-1,3-glucan content by creating several AGS mutants and by incorporating the conditional *alcA* promoter in the CagsB strain. Biochemical analysis of the cell wall of the DagsA, DagsB, DagsA-DagsB, CagsB, and CagsB-DagsA strains revealed that most of the cell wall α-1,3-glucan in *A. nidulans* was synthesized by AgsB under our experimental conditions and that the cell wall β-1,3-glucan and chitin composition was not markedly affected by the loss of cell wall α-1,3-glucan in the *agsB* mutants. This suggests that cell wall α-1,3-glucan is not responsible for fungal viability, but that it seems to protect the cell wall from some cell wall stresses, such as cell wall–degrading enzymes and environmental chemicals. It may also play a role in aggregation of hyphae, particularly in liquid media.

## Supporting Information

Figure S1 **Construction of the** ***agsA*** **gene disruption strains in** ***Aspergillus nidulans.*** (**A**) Schematic illustration of *agsA* gene disruption. The first round of PCR was done to amplify the fragments containing the right and left arms and the selectable marker for the disruption cassette. The second round of PCR was done to fuse the three separate fragments from the first round of PCR. The resulting disruption cassette was used for fungal transformation. Primer agsA-F (Table S1) was derived from the sequences of non-coding regions of *A. nidulans agsA* outside the disruption cassette. Primer agsA-R (Table S1) is specific for the *A. nidulans agsA* coding region. The restriction enzyme sites and the point at which the probes hybridized are indicated. (**B**) PCR results for *agsA* gene disruption in *A. nidulans*. Lane M, λ/*Sty*I digest (molecular weight marker); lane C, control strain (ABPU1); lanes 1–3, DagsA strains (three independently isolated mutant strains). (**C**) Southern analysis of the *agsA* locus in the control and disruption (DagsA) strains using the probe indicated in (**A**). Chromosomal DNA of the control strain (lane C) and of the DagsA strains (lanes 1, 2, and 3) was digested with *Hin*dIII.
(TIF)

Figure S2 **Construction of the** ***agsB*** **gene disruption strains in** ***Aspergillus nidulans.*** (**A**) Schematic illustration of *agsB* gene disruption. The first round of PCR was done to amplify the fragments containing the right and left arms and the selectable marker for the disruption cassette. The second round of PCR was done to fuse the three separate fragments from the first round of PCR. The resulting disruption cassette was used for fungal transformation. Primer agsB-F (Table S1) was derived from the sequences of non-coding regions of *A. nidulans agsB* outside the disruption cassette. Primer agsB-R1 (Table S1) is specific for the *A. nidulans agsB* coding region. The restriction enzyme sites and the point at which the probes hybridized are indicated. (**B**) PCR results for *agsB* gene disruption in *A. nidulans*. Lane M, λ/*Sty*I digest (molecular weight marker); lane C, control strain (ABPU1); lanes 1 and 2, *agsB* disruption strains (two independently isolated mutant strains). (**C**) Southern analysis of the *agsB* locus in the control and *agsB* disruption (DagsB) strains using the probe indicated in (**A**). Chromosomal DNA of the control strain (lanes C) and of the DagsB strains (lanes 1 and 2) was digested with *Sph*I.
(TIF)

Figure S3 **Construction of the conditional-** ***agsB*** **(CagsB) strains in** ***Aspergillus nidulans*** **.** (**A**) Schematic illustration of conditional-*agsB* gene construction in *Aspergillus nidulans.* First line, the control (wild-type) gene; second line, the gene replacement cassette; third line, the conditional-*agsB* gene. Primers PalcA-F and agsB-R2 (Table S1) are specific for the *A. nidulans alcA* promoter and *agsB* coding regions, respectively. The restriction enzyme sites and the point at which the probes hybridized are indicated. (**B**) PCR results for the conditional-*agsB* gene mutation in *A. nidulans*. Lane M, λ/*Sty*I digest (molecular weight marker); lane C, control strain (ABPU1); lane 05, CagsB strain (CagsB05); lane 06, CagsB strain (CagsB06); lane 07, CagsB strain (CagsB07). (**C**) Southern analysis of the *agsB* locus in the control and CagsB strains using the probe indicated in (**A**). Chromosomal DNAs of the control strain (lane C) and the CagsB strains (lanes 05, 06, and 07) were digested with *Bam*HI.
(TIF)

Figure S4 **Fractionation scheme for the** ***A. nidulans*** **cell wall using alkali.** Centrifugation steps were performed at 10,000×*g* for 15 min. Neutralization was done with 17 M acetic acid. HW, AS, and AI indicate the hot-water-soluble, alkali-soluble, and alkali-insoluble fractions, respectively.
(TIF)

Figure S5 **Phenotypic analysis of the** ***agsA*** **disruption strain (DagsA).** (**A**) Expression of *agsA* in the control (CNT) and DagsA strains cultured in CD medium. Conidia (final concentration, 5×10^5^/mL) of the CNT and DagsA strains were inoculated into the indicated liquid medium and cultured for 24 h. RNA samples from each strain were prepared, and the expression of *agsA* was quantified by means of RT-PCR. Each value represents the ratio of the expression relative to the histone H2B gene in each strain. Error bars represent the standard error of the mean calculated for three replicates. (**B**) Hyphal morphology of the control (CNT) and DagsA strains grown in CD liquid medium for 24 h at 37°C. (**C**) Number of conidia of the control (CNT) and DagsA strains obtained from the colonies after 1 week of growth on CD medium at 37°C. Error bars represent the standard deviations (*n* = 3). None of the differences were statistically significant. (**D**) Sensitivities to cell wall–stress compounds in the control (CNT) and DagsA strains. Growth rate (% of the control’s growth) was measured after 4 days of growth on CD medium containing the indicated compound. Left panel, micafungin; center panel, calcofluor white (CFW); right panel, Congo Red. Error bars represent the standard deviations (*n* = 3). None of the differences were statistically significant. (**E**) Susceptibility to Lysing Enzymes of mycelia cultured in CD liquid medium. Mycelia cultured in CD medium for 24 h (30 mg fresh weight) were digested in reaction buffer (10 mM phosphate buffer, pH 6.0) containing 10 mg/mL Lysing Enzymes. After 1, 2, and 4 h of incubation at 30°C, the number of protoplasts in each sample was determined using a hemocytometer. Error bars represent the standard deviations (*n* = 3). None of the differences were statistically significant.
(TIF)

Figure S6 **Phenotypes of the** ***agsB*** **disruption (CagsB) strains.** (**A**) Expression of *agsB* in the control (CNT) and CagsB strains cultured in CD medium (*agsB-*repressing conditions; left) and CDTF medium (*agsB-*inducing conditions; right). Conidia (final concentration, 5×10^5^/mL) of the CNT and CagsB strains were inoculated into the indicated liquid medium and cultured for 24 h. RNA samples of each strain were prepared, and expression of the *agsB* gene was quantified by means of RT-PCR analysis. Each value represents the ratio of expression relative to the histone H2B gene in each strain. Error bars represent the standard error of the mean calculated for three replicates (*P<0.05, **P<0.01). (**B**) Colonial growth of the control (CNT) and CagsB strains. Conidia (a total of 1×10^3^) of each strain were inoculated on the indicated medium and cultured at 37°C for 4 days. (**C**) Number of conidia of the control and CagsB strains obtained from the colonies after 1 week of growth on CD medium at 37°C. Error bars indicate standard deviations of the mean (*n = *3). None of the differences were statistically significant. (**D**) Growth characteristics of the control (CNT) and CagsB strains in liquid media. Conidia (final concentration, 5×10^5^/mL) of the CNT and CagsB strains were inoculated into the indicated liquid medium and rotated at 160 rpm at 37°C for 18 h.
(TIF)

Figure S7 **Ultrathin sections of the control and CagsB strains of** ***Aspergillus nidulans*** **.** Cells of each strain were cultured in CD liquid medium (CNT_CD for the control and CagsB_CD for the conditional-*agsB* panels) or CDTF liquid medium (CNT_CDTF for the control and CagsB_CDTF for the conditional-*agsB* panels) at 37°C for 24 h, collected by centrifugation, and snap-frozen by plunging into a melting propane/isopentane mixture cooled with liquid nitrogen. Cells were then freeze-substituted in acetone, embedded in resin, sectioned, and examined by transmission electron microscopy. A medial gray band in the cell wall, which might be attributable to alterations in the cell wall components, was sometimes observed in the CagsB strain cultured in CDTF medium. However, no significant differences in the thickness of the cell wall were observed between the control and CagsB strains (Table S2). CW, cell wall; PM, plasma membrane. Scale bar = 500 nm.
(TIF)

Figure S8 **^13^C-NMR spectra of bacterial mutan and of the AS2 fraction from the control strain.** (**A**) The ^13^C-NMR spectrum of mutan predominantly contained six signals (at 101.3, 83.5, 73.6, 71.9, 71.5, and 62.0 ppm) that were attributable to the presence of α-1,3-glucan [32]. (**B**) The ^13^C NMR spectrum of the AS2 fraction from the control strain predominantly contained six signals (at 101.2, 83.4, 73.5, 71.9, 71.4, and 61.9 ppm) that were also attributable to α-1,3-glucan.
(TIF)

Figure S9 **Saccharides liberated from the AS2 and AI fractions after treatment with α-1,3-glucanase or β-1,3-glucanase.** (**A**) The amount of glucose liberated from the AS2 and AI fractions derived from the control (CNT), *agsB* disruption (DagsB), and conditional-*agsB* (CagsB) strains after treatment with purified α-1,3-glucanase from *Bacillus circulans* KA-304 [25]. Error bars represent the standard deviations of the mean (*n* = 3). *, significantly different from the control (*P*<0.01). (**B**) The amount of glucose liberated from the AS2 and AI fractions derived from the CNT, DagsB, and CagsB strains after treatment with the purified β-1,3-glucanase from *A. niger*. Error bars represent the standard deviations (*n* = 3). None of the differences were statistically significant.
(TIF)

Table S1 **PCR primers used in this study.**
(DOCX)

Table S2 **Cell wall thickness of the control (CNT) and conditional-** ***agsB*** **(CagsB) strains.**
(DOCX)

Table S3 **Ratios of methyl (Me) ethers obtained after methanolysis of the permethylated bacterial mutan and the AS2 fraction (see Figure S4) of the control strain in** ***A. nidulans*** **.**
(DOCX)
