# Supplementary figures and images for: Functional Analysis of the α-1,3-Glucan Synthase Genes agsA and agsB in Aspergillus nidulans: AgsB Is the Major α-1,3-Glucan Synthase in This Fungus
Source: PLoS One. 2013 Jan 24;8(1):e54893. doi: 10.1371/journal.pone.0054893 (PMC3554689; doi:10.1371/journal.pone.0054893)

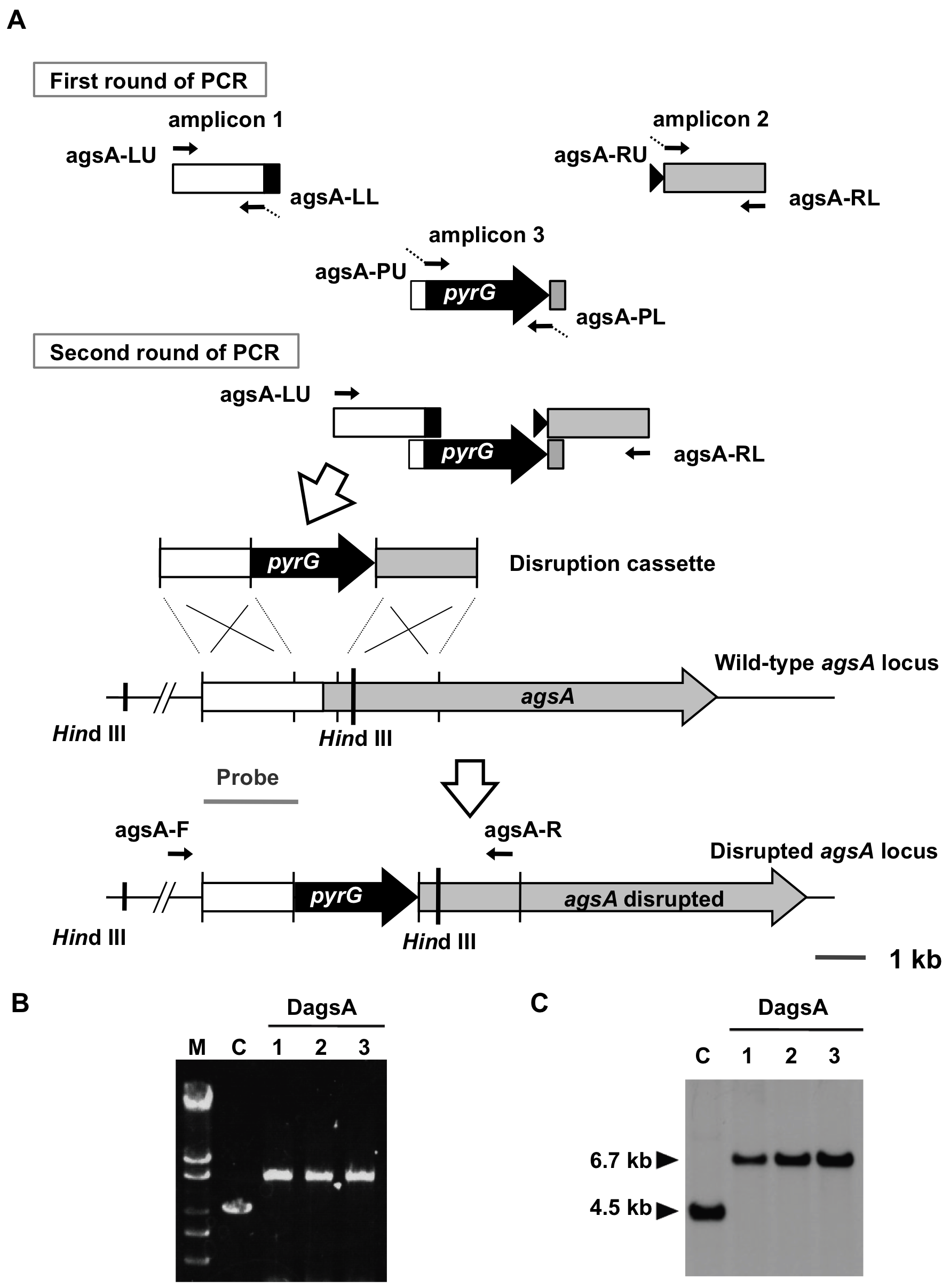

Supplement: Figure S1 — Construction of the agsA gene disruption strains in Aspergillus nidulans. (A) Schematic illustration of agsA gene disruption. The first round of PCR was done to amplify the fragments containing the right and left arms and the selectable marker for the disruption cassette. The second round of PCR was done to fuse the three separate fragments from the first round of PCR. The resulting disruption cassette was used for fungal transformation. Primer agsA-F (Table S1) was derived from the sequences of non-coding regions of A. nidulans agsA outside the disruption cassette. Primer agsA-R (Table S1) is specific for the A. nidulans agsA coding region. The restriction enzyme sites and the point at which the probes hybridized are indicated. (B) PCR results for agsA gene disruption in A. nidulans. Lane M, λ/StyI digest (molecular weight marker); lane C, control strain (ABPU1); lanes 1–3, DagsA strains (three independently isolated mutant strains). (C) Southern analysis of the agsA locus in the control and disruption (DagsA) strains using the probe indicated in (A). Chromosomal DNA of the control strain (lane C) and of the DagsA strains (lanes 1, 2, and 3) was digested with HindIII. (TIF) [file pone.0054893.s001.tif]

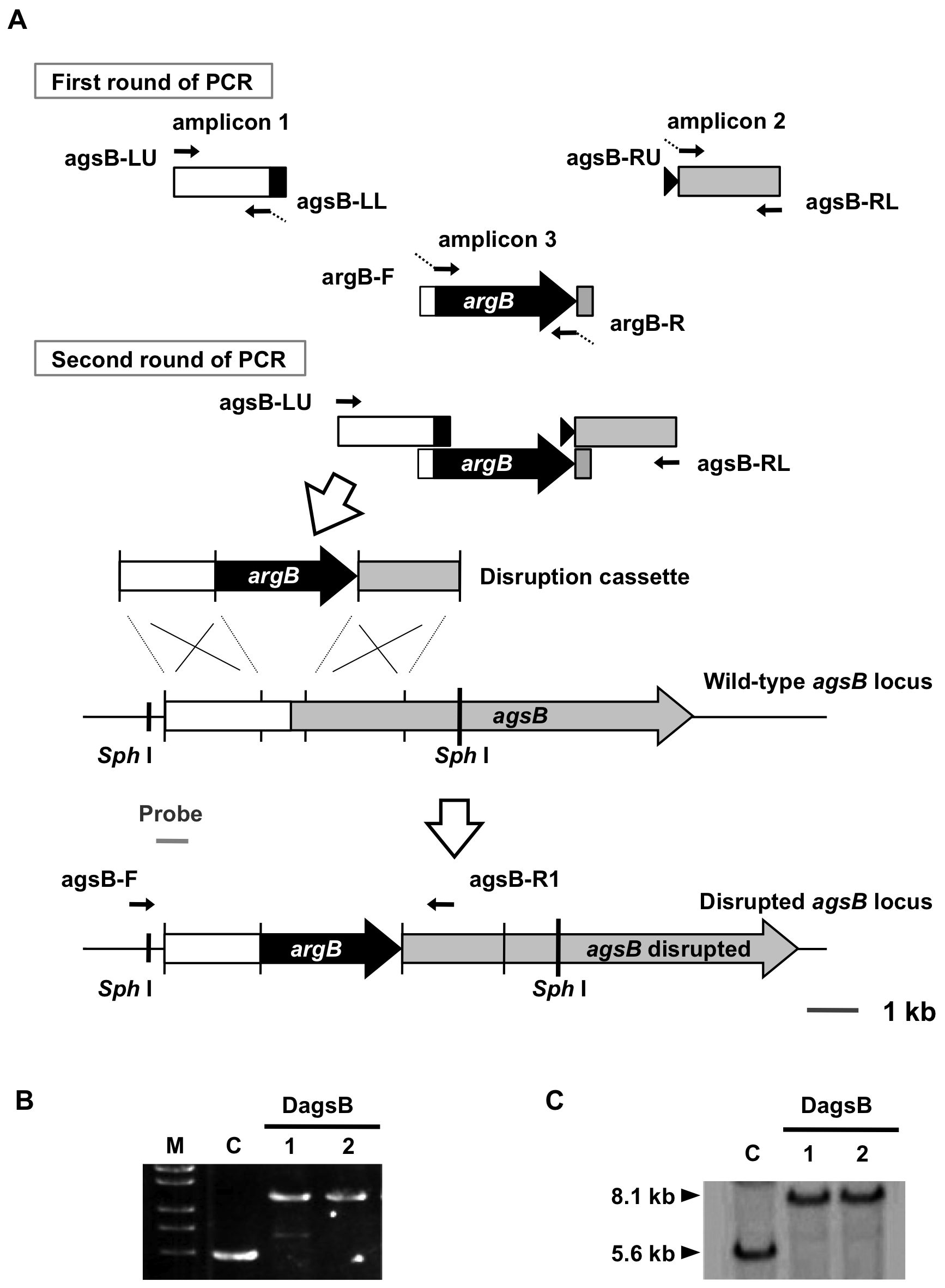

Supplement: Figure S2 — Construction of the agsB gene disruption strains in Aspergillus nidulans. (A) Schematic illustration of agsB gene disruption. The first round of PCR was done to amplify the fragments containing the right and left arms and the selectable marker for the disruption cassette. The second round of PCR was done to fuse the three separate fragments from the first round of PCR. The resulting disruption cassette was used for fungal transformation. Primer agsB-F (Table S1) was derived from the sequences of non-coding regions of A. nidulans agsB outside the disruption cassette. Primer agsB-R1 (Table S1) is specific for the A. nidulans agsB coding region. The restriction enzyme sites and the point at which the probes hybridized are indicated. (B) PCR results for agsB gene disruption in A. nidulans. Lane M, λ/StyI digest (molecular weight marker); lane C, control strain (ABPU1); lanes 1 and 2, agsB disruption strains (two independently isolated mutant strains). (C) Southern analysis of the agsB locus in the control and agsB disruption (DagsB) strains using the probe indicated in (A). Chromosomal DNA of the control strain (lanes C) and of the DagsB strains (lanes 1 and 2) was digested with SphI. (TIF) [file pone.0054893.s002.tif]

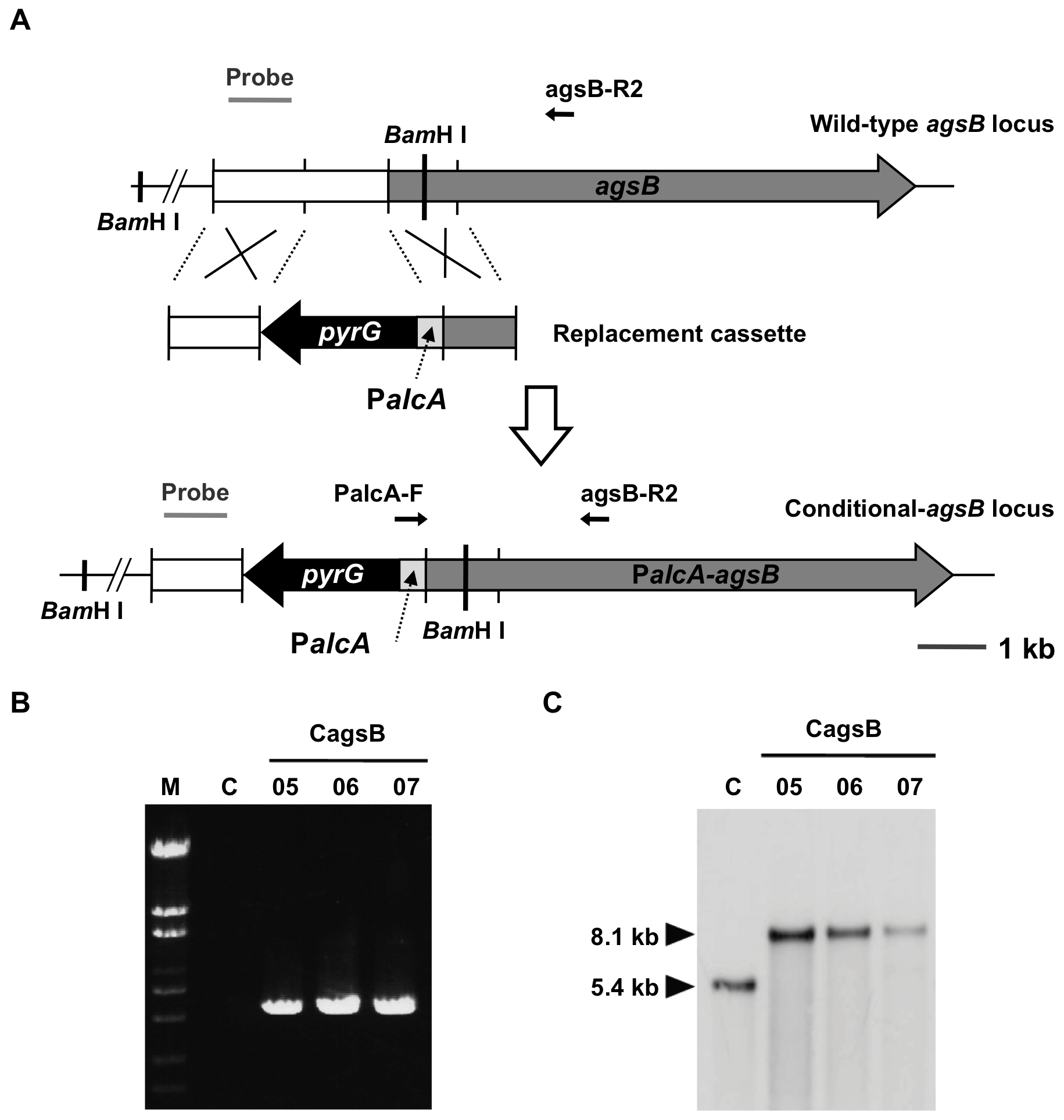

Supplement: Figure S3 — Construction of the conditional- agsB (CagsB) strains in Aspergillus nidulans . (A) Schematic illustration of conditional-agsB gene construction in Aspergillus nidulans. First line, the control (wild-type) gene; second line, the gene replacement cassette; third line, the conditional-agsB gene. Primers PalcA-F and agsB-R2 (Table S1) are specific for the A. nidulans alcA promoter and agsB coding regions, respectively. The restriction enzyme sites and the point at which the probes hybridized are indicated. (B) PCR results for the conditional-agsB gene mutation in A. nidulans. Lane M, λ/StyI digest (molecular weight marker); lane C, control strain (ABPU1); lane 05, CagsB strain (CagsB05); lane 06, CagsB strain (CagsB06); lane 07, CagsB strain (CagsB07). (C) Southern analysis of the agsB locus in the control and CagsB strains using the probe indicated in (A). Chromosomal DNAs of the control strain (lane C) and the CagsB strains (lanes 05, 06, and 07) were digested with BamHI. (TIF) [file pone.0054893.s003.tif]

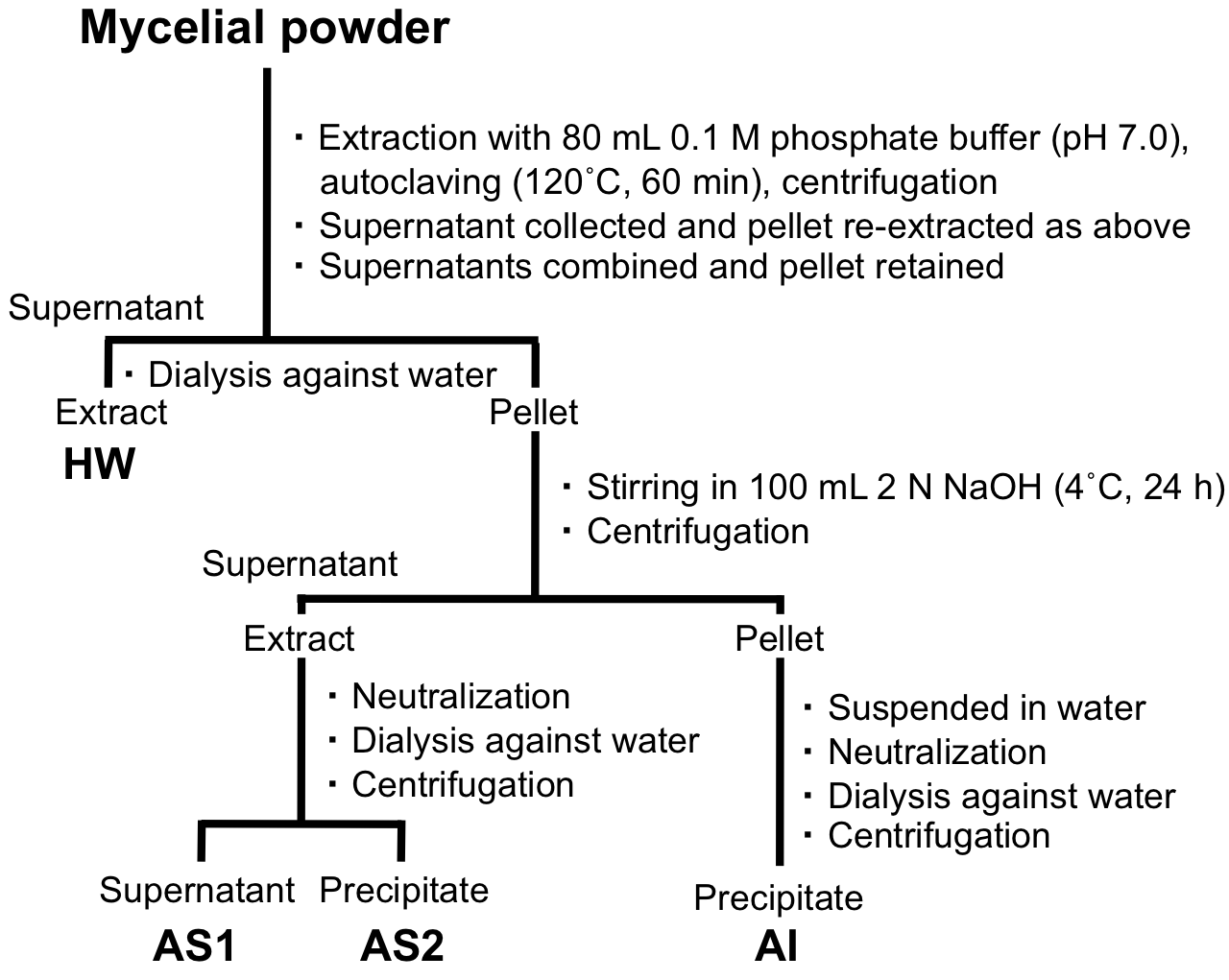

Supplement: Figure S4 — Fractionation scheme for the A. nidulans cell wall using alkali. Centrifugation steps were performed at 10,000×g for 15 min. Neutralization was done with 17 M acetic acid. HW, AS, and AI indicate the hot-water-soluble, alkali-soluble, and alkali-insoluble fractions, respectively. (TIF) [file pone.0054893.s004.tif]

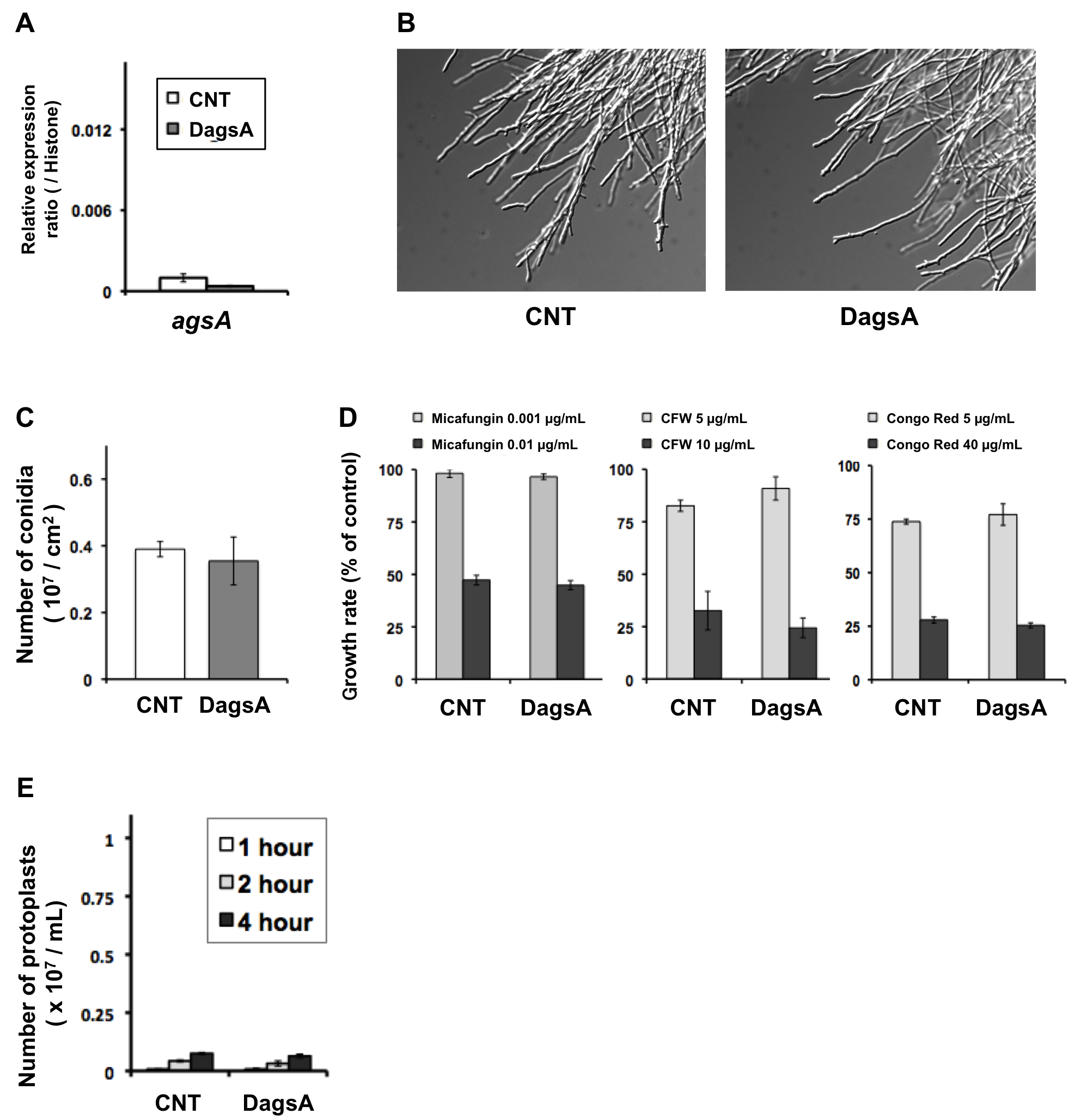

Supplement: Figure S5 — Phenotypic analysis of the agsA disruption strain (DagsA). (A) Expression of agsA in the control (CNT) and DagsA strains cultured in CD medium. Conidia (final concentration, 5×10^5^/mL) of the CNT and DagsA strains were inoculated into the indicated liquid medium and cultured for 24 h. RNA samples from each strain were prepared, and the expression of agsA was quantified by means of RT-PCR. Each value represents the ratio of the expression relative to the histone H2B gene in each strain. Error bars represent the standard error of the mean calculated for three replicates. (B) Hyphal morphology of the control (CNT) and DagsA strains grown in CD liquid medium for 24 h at 37°C. (C) Number of conidia of the control (CNT) and DagsA strains obtained from the colonies after 1 week of growth on CD medium at 37°C. Error bars represent the standard deviations (n = 3). None of the differences were statistically significant. (D) Sensitivities to cell wall–stress compounds in the control (CNT) and DagsA strains. Growth rate (% of the control’s growth) was measured after 4 days of growth on CD medium containing the indicated compound. Left panel, micafungin; center panel, calcofluor white (CFW); right panel, Congo Red. Error bars represent the standard deviations (n = 3). None of the differences were statistically significant. (E) Susceptibility to Lysing Enzymes of mycelia cultured in CD liquid medium. Mycelia cultured in CD medium for 24 h (30 mg fresh weight) were digested in reaction buffer (10 mM phosphate buffer, pH 6.0) containing 10 mg/mL Lysing Enzymes. After 1, 2, and 4 h of incubation at 30°C, the number of protoplasts in each sample was determined using a hemocytometer. Error bars represent the standard deviations (n = 3). None of the differences were statistically significant. (TIF) [file pone.0054893.s005.tif]

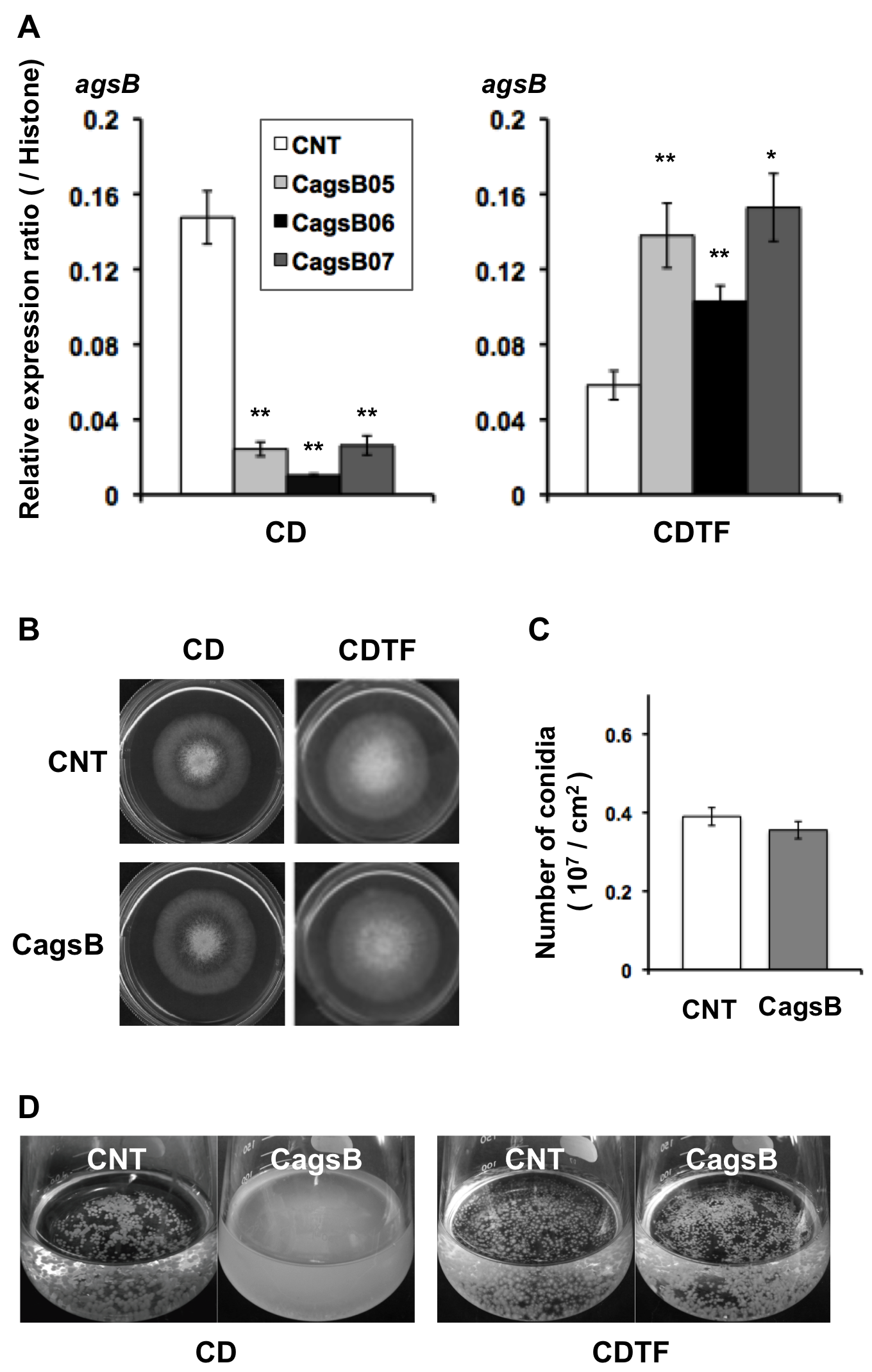

Supplement: Figure S6 — Phenotypes of the agsB disruption (CagsB) strains. (A) Expression of agsB in the control (CNT) and CagsB strains cultured in CD medium (agsB-repressing conditions; left) and CDTF medium (agsB-inducing conditions; right). Conidia (final concentration, 5×10^5^/mL) of the CNT and CagsB strains were inoculated into the indicated liquid medium and cultured for 24 h. RNA samples of each strain were prepared, and expression of the agsB gene was quantified by means of RT-PCR analysis. Each value represents the ratio of expression relative to the histone H2B gene in each strain. Error bars represent the standard error of the mean calculated for three replicates (*P<0.05, **P<0.01). (B) Colonial growth of the control (CNT) and CagsB strains. Conidia (a total of 1×10^3^) of each strain were inoculated on the indicated medium and cultured at 37°C for 4 days. (C) Number of conidia of the control and CagsB strains obtained from the colonies after 1 week of growth on CD medium at 37°C. Error bars indicate standard deviations of the mean (n = 3). None of the differences were statistically significant. (D) Growth characteristics of the control (CNT) and CagsB strains in liquid media. Conidia (final concentration, 5×10^5^/mL) of the CNT and CagsB strains were inoculated into the indicated liquid medium and rotated at 160 rpm at 37°C for 18 h. (TIF) [file pone.0054893.s006.tif]

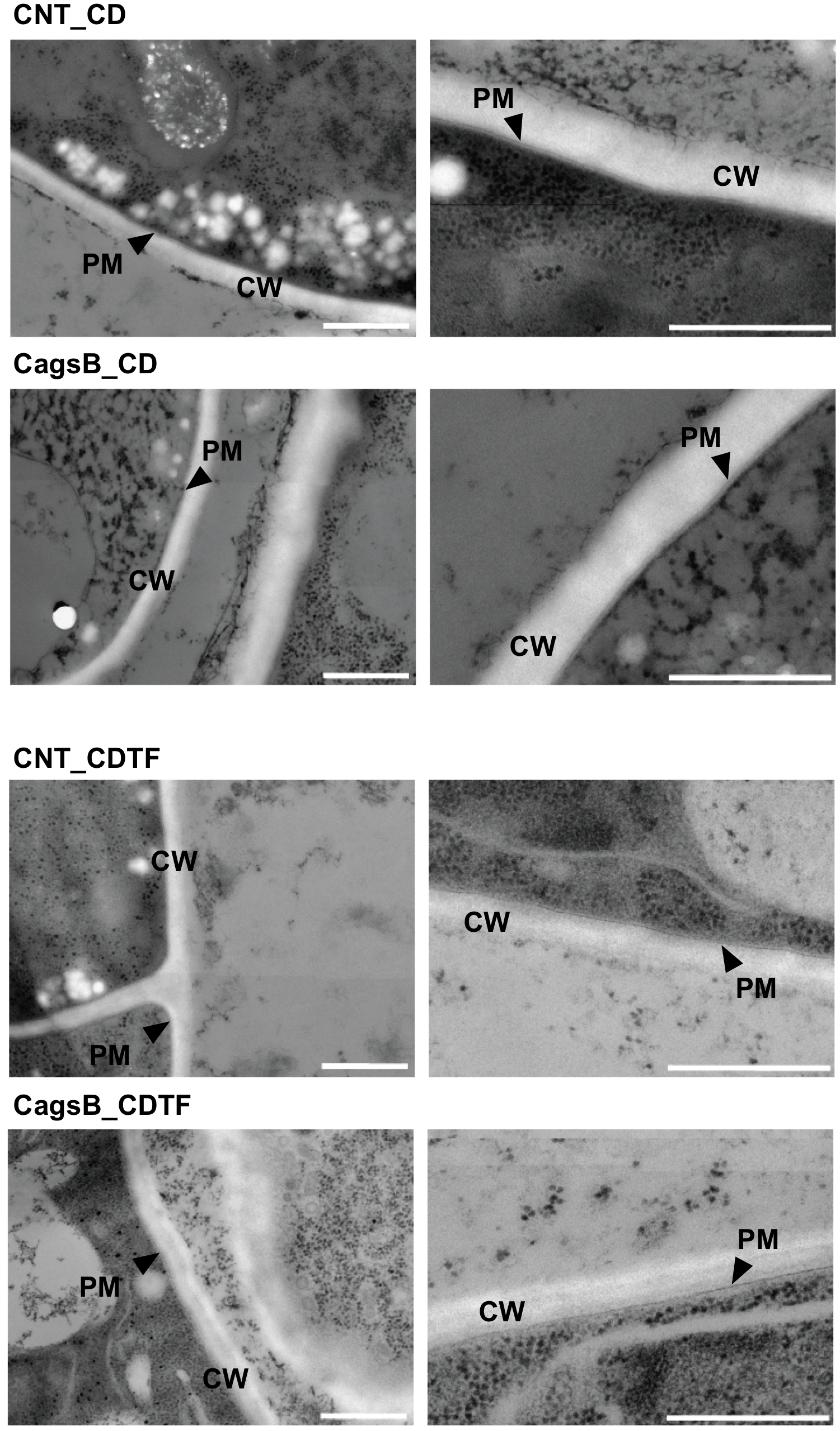

Supplement: Figure S7 — Ultrathin sections of the control and CagsB strains of Aspergillus nidulans . Cells of each strain were cultured in CD liquid medium (CNT_CD for the control and CagsB_CD for the conditional-agsB panels) or CDTF liquid medium (CNT_CDTF for the control and CagsB_CDTF for the conditional-agsB panels) at 37°C for 24 h, collected by centrifugation, and snap-frozen by plunging into a melting propane/isopentane mixture cooled with liquid nitrogen. Cells were then freeze-substituted in acetone, embedded in resin, sectioned, and examined by transmission electron microscopy. A medial gray band in the cell wall, which might be attributable to alterations in the cell wall components, was sometimes observed in the CagsB strain cultured in CDTF medium. However, no significant differences in the thickness of the cell wall were observed between the control and CagsB strains (Table S2). CW, cell wall; PM, plasma membrane. Scale bar = 500 nm. (TIF) [file pone.0054893.s007.tif]

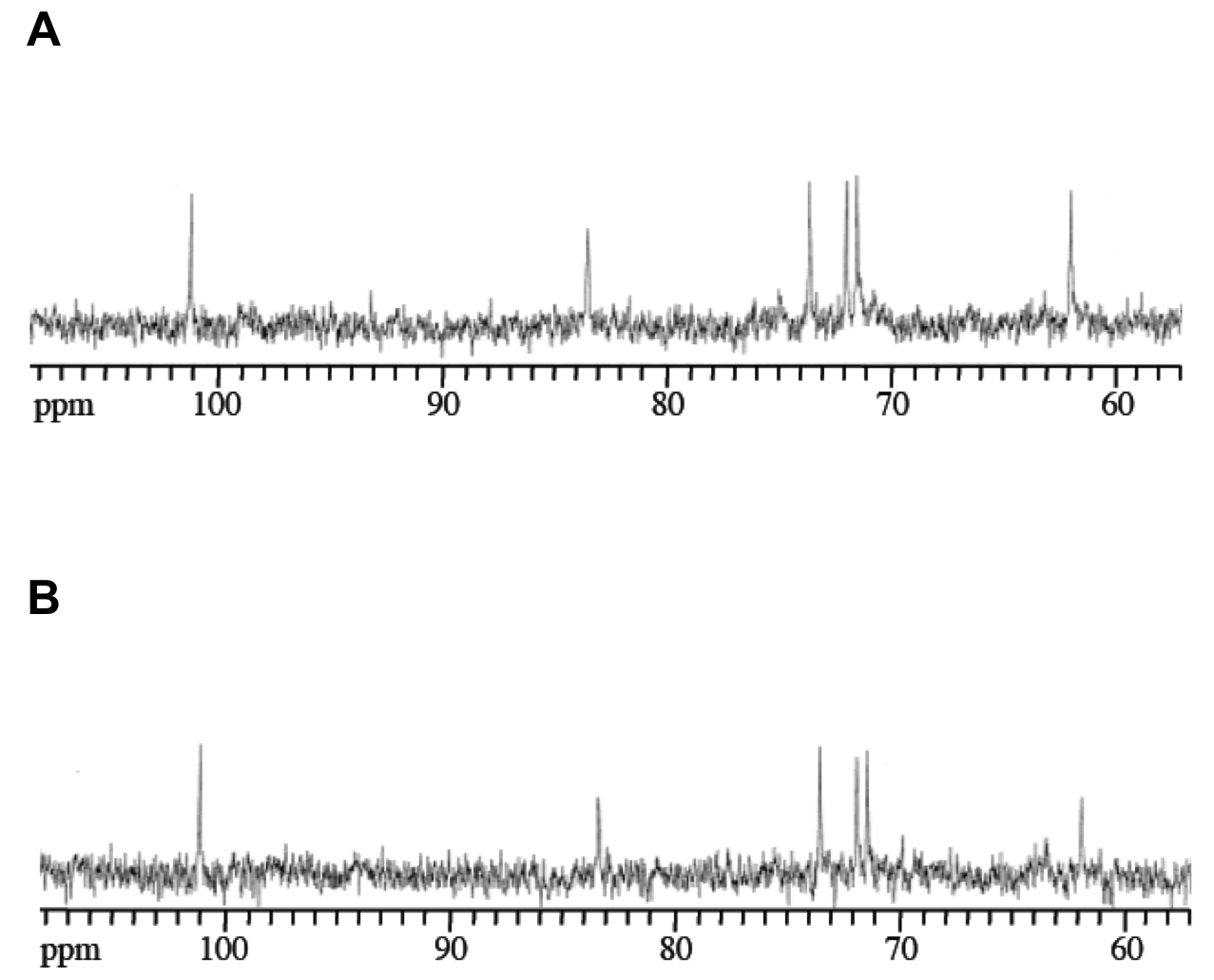

Supplement: Figure S8 — 13C-NMR spectra of bacterial mutan and of the AS2 fraction from the control strain. (A) The 13C-NMR spectrum of mutan predominantly contained six signals (at 101.3, 83.5, 73.6, 71.9, 71.5, and 62.0 ppm) that were attributable to the presence of α-1,3-glucan [32]. (B) The 13C NMR spectrum of the AS2 fraction from the control strain predominantly contained six signals (at 101.2, 83.4, 73.5, 71.9, 71.4, and 61.9 ppm) that were also attributable to α-1,3-glucan. (TIF) [file pone.0054893.s008.tif]

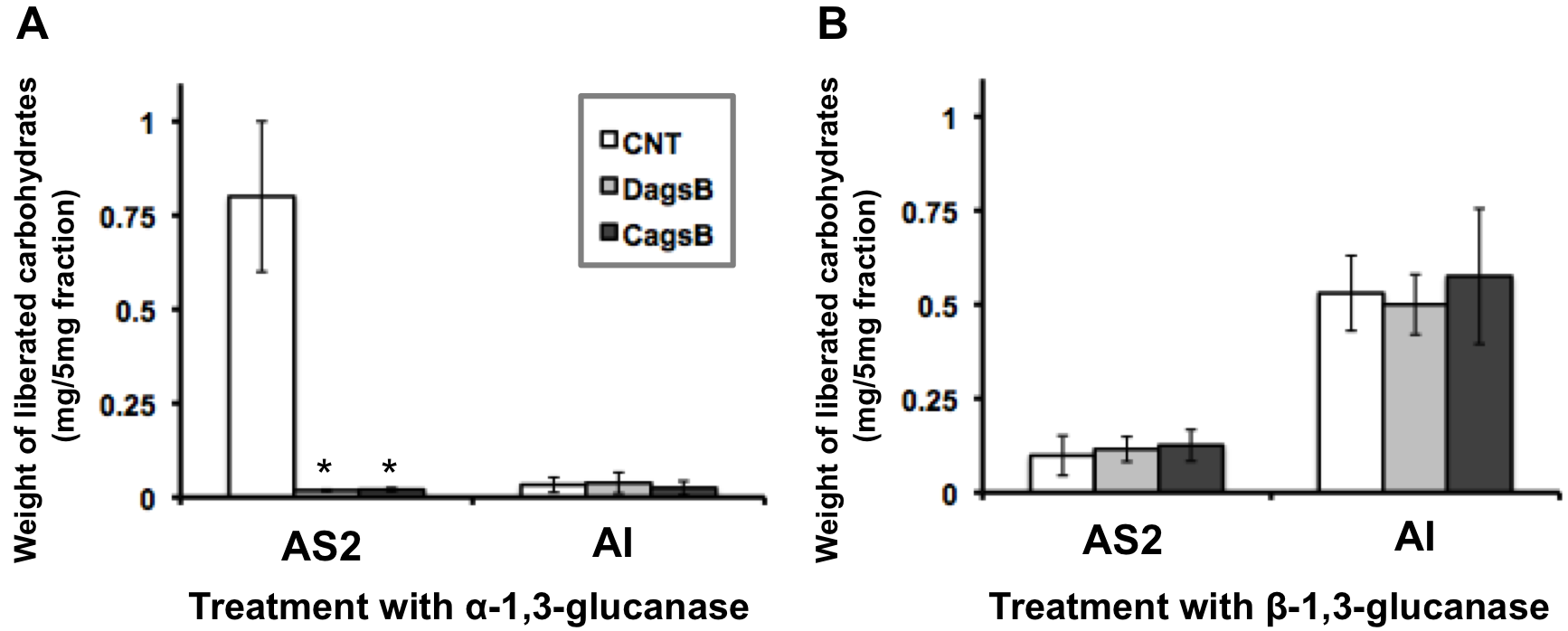

Supplement: Figure S9 — Saccharides liberated from the AS2 and AI fractions after treatment with α-1,3-glucanase or β-1,3-glucanase. (A) The amount of glucose liberated from the AS2 and AI fractions derived from the control (CNT), agsB disruption (DagsB), and conditional-agsB (CagsB) strains after treatment with purified α-1,3-glucanase from Bacillus circulans KA-304 [25]. Error bars represent the standard deviations of the mean (n = 3). *, significantly different from the control (P<0.01). (B) The amount of glucose liberated from the AS2 and AI fractions derived from the CNT, DagsB, and CagsB strains after treatment with the purified β-1,3-glucanase from A. niger. Error bars represent the standard deviations (n = 3). None of the differences were statistically significant. (TIF) [file pone.0054893.s009.tif]
