# Supplementary material for: Functional Analysis of the α-1,3-Glucan Synthase Genes agsA and agsB in Aspergillus nidulans: AgsB Is the Major α-1,3-Glucan Synthase in This Fungus
Source: PLoS One. 2013 Jan 24;8(1):e54893. doi: 10.1371/journal.pone.0054893 (PMC3554689; doi:10.1371/journal.pone.0054893)
Supplement: Table S1 — PCR primers used in this study. (DOCX) [file pone.0054893.s010.docx]

|  | Table S1.　PCR primers used in this study | | | | | | | | | |  |  |  |
| --- | --- | --- | --- | --- | --- | --- | --- | --- | --- | --- | --- | --- | --- |
|  |  |  |  | |  | |  | | | |  |  |  |
|  |  |  |  | |  | |  | | | |  |  |  |
|  | Purpose |  | Name | |  | | Sequence (5'−3') | | | |  |  |  |
|  |  |  |  | |  | |  | | | |  |  |  |
|  |  |  |  | |  | |  | | | |  |  |  |
|  | PCR for *agsA* disruption | | | |  | |  | | | |  |  |  |
|  |  |  |  | |  | |  | | | |  |  |  |
|  |  |  | agsA-LU | |  | | AGTGGAGGAGTTAGGGAGTGAT | | | |  |  |  |
|  |  |  | agsA-LL | |  | | CACAGGGTACGTCTGTTGTGAAAGAGTAAGGTAGAAGCCCC | | | |  |  |  |
|  |  |  | agsA-RU | |  | | TTCTTCTGAGGTGCAGTTCAGCAGATTATTACGCACCGGA | | | |  |  |  |
|  |  |  | agsA-RL | |  | | AACCGTGGTTTTGGTGGCAAAG | | | |  |  |  |
|  |  |  | agsA-PU | |  | | TACCTTACTCTTTCACAACAGACGTACCCTGTGATGTTC | | | |  |  |  |
|  |  |  | agsA-PL | |  | | GTAATAATCTGCTGAACTGCACCTCAGAAGAAAAGGATG | | | |  |  |  |
|  |  |  | agsA-F | |  | | GTACGGTGTAAGCTGCTCGCTGGAC | | | |  |  |  |
|  |  |  | agsA-R | |  | | TCCTGGATCTTGTAAACTGAGTCTC | | | |  |  |  |
|  |  |  |  | |  | |  | | | |  |  |  |
|  | PCR for *agsB* disruption | | | |  | |  | | | |  |  |  |
|  |  |  |  | |  | |  | | | |  |  |  |
|  |  |  | agsB-LU | |  | | GCAATGAGAGCTGGAATCAGTG | | | |  |  |  |
|  |  |  | agsB-LL | |  | | TGAGTCGGCCACAGCGGATGGAATTCGTCGTCTGGCTGTGAGTGTAAC | | | |  |  |  |
|  |  |  | agsB-RU | |  | | TCTTCCAGTTACTCCGTCGGTACCCAGCAACATGCTGGCCATACGAC | | | |  |  |  |
|  |  |  | agsB-RL | |  | | AAAGTCCTGGGTCTCTTCGTTC | | | |  |  |  |
|  |  |  | argB-F | |  | | GAATTCCATCCGCTGTGGCCGACTCA | | | |  |  |  |
|  |  |  | argB-R | |  | | GGTACCGACGGAGTAACTGGAAAGATACGA | | | |  |  |  |
|  |  |  | agsB-F | |  | | AGGAAAGACTGTTGGATGAG | | | |  |  |  |
|  |  |  | agsB-R1 | |  | | GACTTATTCGTGTTGACGTTGTA | | | |  |  |  |
|  |  |  |  | |  | |  | | | |  |  |  |
|  | PCR for complementation of *agsB* disruptant | | | |  | |  | | | |  |  |  |
|  |  |  | agsB-T400R  -HindIII | |  | | GGTAGTTGAAAGCTTGGTACTTTAATCCAAAAGAG | | | |  |  |  |
|  | | | | | | | |  |  |  |  |  |  |
|  | PCR for conditional-*agsB* (CagsB) construction | | |  | |  | | |  |  | | |  |
|  |  |  | CagsB-LU | |  | | AGGAAAGACTGTTGGATGAG | | | |  |  |  |
|  |  |  | CagsB-LL | |  | | AGACAAGCACTGAGAGTACTCCGTACAGCTATG | | | |  |  |  |
|  |  |  | CagsB-RU | |  | | ATCACCTCGCCTCAAAATGGGGAGGCTCCAGCTCTCAA | | | |  |  |  |
|  |  |  | CagsB-RL | |  | | CCGTACAGGTGGGTACCATTCTCTC | | | |  |  |  |
|  |  |  | CagsB-PU | |  | | ATAGCTGTACGGAGTACTCTCAGTGCTTGTCTACCAGATTAG | | | |  |  |  |
|  |  |  | CagsB-PL | |  | | GAGAGCTGGAGCCTCCCCATTTTGAGGCGAGGTGATAGGATTG | | | |  |  |  |
|  |  |  | PalcA-F | |  | | CGGGATAGTTCCGACCTAGGATTGGATGCA | | | |  |  |  |
|  |  |  | agsB-R2 | |  | | CAGTGATGTTCCCACACTGGAC | | | |  |  |  |
|  |  |  |  | |  | |  | | | |  |  |  |
|  | PCR for agsA disruption in the CagsB strain | | | |  | |  | | | |  |  |  |
|  |  |  |  | |  | |  | | | |  |  |  |
|  |  |  | agsA-F | |  | | GTACGGTGTAAGCTGCTCGCTGGAC | | | |  |  |  |
|  |  |  | agsA-LL2 | |  | | TGTTTCGGTTGAGTCGGCCACAGCGGATGGAATTCCTGTATGTAGCGGTACGCATTGAAC | | | |  |  |  |
|  |  |  | agsA-RU2 | |  | | AAAGCTCGTATCTTTCCAGTTACTCCGTCGGTACCCTCCGTGAATGGAACCCAAGCGTTC | | | |  |  |  |
|  |  |  | agsA-R | |  | | TCCTGGATCTTGTAAACTGAGTCTC | | | |  |  |  |
|  |  |  | argB-F | |  | | GAATTCCATCCGCTGTGGCCGACTCA | | | |  |  |  |
|  |  |  | argB-R | |  | | GGTACCGACGGAGTAACTGGAAAGATACGA | | | |  |  |  |
|  |  |  |  | |  | |  | | | |  |  |  |
|  | Primers for quantitative-RT PCR | | | |  | |  | | | |  |  |  |
|  |  |  |  | |  | |  | | | |  |  |  |
|  |  |  | agsA-RT-F | |  | | GCTTTCCAAATCCCACAGTTGG | | | |  |  |  |
|  |  |  | agsA-RT-R | |  | | GTGAAGCAGATATGCATCCGTG | | | |  |  |  |
|  |  |  | agsB-RT-F | |  | | ATCGGACACTACCTTCCCTG | | | |  |  |  |
|  |  |  | agsB-RT-R | |  | | GACTTGGCTGACGATCAACG | | | |  |  |  |
|  |  |  | chsA-RT-F | |  | | TGCAGTACGGACGTATATGG | | | |  |  |  |
|  |  |  | chsA-RT-R | |  | | CAGATACAGAACTGCATACGC | | | |  |  |  |
|  |  |  | chsB-RT-F | |  | | CTTGAACGTTTACGCCTTCAGC | | | |  |  |  |
|  |  |  | chsB-RT-R | |  | | TCGTCCAGACTCTTCTCTTCC | | | |  |  |  |
|  |  |  | chsC-RT-F | |  | | GCGATGTGGTTTTTGGTTGTGC | | | |  |  |  |
|  |  |  | chsC-RT-R | |  | | CATTGCGATATGCTGAACCTGC | | | |  |  |  |
|  |  |  | chsD-RT-F | |  | | ATGGAGCTGGTCTTGGTTCG | | | |  |  |  |
|  |  |  | chsD-RT-R | |  | | CCAAGAATAAGGGCGAGCAAG | | | |  |  |  |
|  |  |  | csmA-RT-F | |  | | CCGACGAAGGAAAATTCGACC | | | |  |  |  |
|  |  |  | csmA-RT-R | |  | | GAGACATCCGAGACATATGCC | | | |  |  |  |
|  |  |  | csmB-RT-F | |  | | ACAGATAACCTTCTTCGACC | | | |  |  |  |
|  |  |  | csmB-RT-R | |  | | CGTCGTCTGAAGTCGTTGTTC | | | |  |  |  |
|  |  |  | fksA-RT-F | |  | | CTCAGCAGACTTCGTCATTGG | | | |  |  |  |
|  |  |  | fksA-RT-R | |  | | CAGAATAGCGAAACGGACCAC | | | |  |  |  |
|  |  |  | gelA-RT-F | |  | | CTACGGTCTTCCCCTTTTCC | | | |  |  |  |
|  |  |  | gelA-RT-R | |  | | GCTCAGTAACCTTGCCGTTC | | | |  |  |  |
|  |  |  | gelB-RT-F | |  | | TCGTCGACTTTGACAACCTGC | | | |  |  |  |
|  |  |  | gelB-RT-R | |  | | TGATCAAGTCCTGGACACCAG | | | |  |  |  |
|  |  |  | gfaA-RT-F | |  | | TCCTATCTGCACTGTGAAGCC | | | |  |  |  |
|  |  |  | gfaA-RT-R | |  | | CGTTGAGAAGTCCTTGAAGGC | | | |  |  |  |
|  |  |  | Histone-RT-F | |  | | CACCCGGACACTGGTATCTC | | | |  |  |  |
|  |  |  | Histone-RT-R | |  | | GAATACTTCGTAACGGCCTTGG | | | |  |  |  |
|  |  |  |  | |  | |  | | | |  |  |  |
