# Supplementary material for: Functional Analysis of the α-1,3-Glucan Synthase Genes agsA and agsB in Aspergillus nidulans: AgsB Is the Major α-1,3-Glucan Synthase in This Fungus
Source: PLoS One. 2013 Jan 24;8(1):e54893. doi: 10.1371/journal.pone.0054893 (PMC3554689; doi:10.1371/journal.pone.0054893)
Supplement: Table S2 — Cell wall thickness of the control (CNT) and conditional- agsB (CagsB) strains. (DOCX) [file pone.0054893.s011.docx]

|  | Table S2. Cell wall thickness of the control (CNT) and conditional-*agsB* (CagsB) strains. | | | | |  |
| --- | --- | --- | --- | --- | --- | --- |
|  |  |  |  |  |  |  |
|  |  |  | Cell wall thickness (nm) | | | |
|  |  |  |  |  |  |  |
|  |  |  |  |  |  |  |
|  | Strain |  | CD medium (*agsB*-repressing) |  | CDTF medium (*agsB*-inducing) |  |
|  |  |  |  |  |  |  |
|  |  |  |  |  |  |  |
|  | CNT |  | 127.78 ± 18.94 |  | 107.42 ± 20.14 |  |
|  |  |  |  |  |  |  |
|  | CagsB |  | 134.18 ± 19.84 |  | 127.62 ± 43.05 |  |
|  |  |  |  |  |  |  |
|  |  |  |  |  |  |  |
|  | For each measurement, the mean value ± SD is indicated. No differences were statistically significant | | | | |  |
