# Supplementary material for: Functional Analysis of the α-1,3-Glucan Synthase Genes agsA and agsB in Aspergillus nidulans: AgsB Is the Major α-1,3-Glucan Synthase in This Fungus
Source: PLoS One. 2013 Jan 24;8(1):e54893. doi: 10.1371/journal.pone.0054893 (PMC3554689; doi:10.1371/journal.pone.0054893)
Supplement: Table S3 — Ratios of methyl (Me) ethers obtained after methanolysis of the permethylated bacterial mutan and the AS2 fraction (see Figure S4) of the control strain in A. nidulans . (DOCX) [file pone.0054893.s012.docx]

| Table S3. Ratios of methyl (Me) ethers obtained after methanolysis of the permethylated bacterial mutan and the AS2 fraction (see Figure S4) of the control strain in *A. nidulans*. | | | | | | | | | | | | | | | |
| --- | --- | --- | --- | --- | --- | --- | --- | --- | --- | --- | --- | --- | --- | --- | --- |
|  |  |  |  |  |  |  |  |  |  | |  |  | | |  |
|  |  |  |  |  |  |  |  |  |  | |  |  | | |  |
|  |  |  |  |  |  |  | Molar ratios (%)^b^ | | | | | | | |  |
|  |  |  |  |  |  |  |  | |  |  | | |  |  |  |
|  | Alditol acetates^a^ |  | Linkages |  | Retention  time (min.) |  | Pachyman (a β-1,3-glucan) | |  | Bacterial  mutan | | |  | CNT  AS2 |  |
|  |  |  |  |  |  |  |  | |  |  | | |  |  |  |
|  |  |  |  |  |  |  |  | |  |  | | |  |  |  |
|  | 2,3,4,6-tetra-*O*-Me-Glc |  | G_1-_ |  | 10.3 |  | trace | |  | less than 0.4 | | |  | 0.5 |  |
|  |  |  |  |  |  |  |  | |  |  | | |  |  |  |
|  | 2,4,6-tri-*O*-Me-Glc |  | _-3_G_1-_ |  | 13.6 |  | 100 | |  | 99.5 | | |  | 98.5 |  |
|  |  |  |  |  |  |  |  | |  |  | | |  |  |  |
|  | 2,3,4-tri-*O*-Me-Glc |  | ^-6^G_1-_ |  | 14.4 |  | N. D. | |  | N. D. | | |  | 0.5 |  |
|  |  |  |  |  |  |  |  | |  |  | | |  |  |  |
|  | 2,4-di-*O*-Me-Glc |  | ^-^_-_^6^_3_G_1-_ |  | 21.2 |  | N. D. | |  | N. D. | | |  | less than 0.4 |  |
|  |  |  |  |  |  |  |  | |  |  | | |  |  |  |
|  |  |  |  |  |  |  |  | |  |  | | |  |  |  |
|  | ^a^ 2,3,4,6-tetra-*O*-Me-Glc = 1,5-diacetyl-2,3,4,6,-tetra-*O*-methyl-D-glucitol, and so on. | | | | | | | | | | | | | |  |
|  | ^b^ trace, a small amount of the methyl ester derivative was detected; N. D., not detected | | | | | | | | | | | | | |  |
|  |  |  |  |  |  |  |  | |  |  | | |  |  |  |
